# Genetic algorithm based hybrid approach to solve fuzzy multi-objective assignment problem using exponential membership function

**DOI:** 10.1186/s40064-016-3685-0

**Published:** 2016-11-28

**Authors:** Jayesh M. Dhodiya, Anita Ravi Tailor

**Affiliations:** Department of Applied Mathematics and Humanities, S. V. National Institute of Technology, Surat, 395007 India

**Keywords:** Multi-objective assignment problem, *α*-Level sets, Fuzzy membership function, Triangular fuzzy number

## Abstract

This paper presents a genetic algorithm based hybrid approach for solving a fuzzy multi-objective assignment problem (FMOAP) by using an exponential membership function in which the coefficient of the objective function is described by a triangular possibility distribution. Moreover, in this study, fuzzy judgment was classified using *α*-level sets for the decision maker (DM) to simultaneously optimize the optimistic, most likely, and pessimistic scenarios of fuzzy objective functions. To demonstrate the effectiveness of the proposed approach, a numerical example is provided with a data set from a realistic situation. This paper concludes that the developed hybrid approach can manage FMOAP efficiently and effectively with an effective output to enable the DM to take a decision.

## Background

The assignment problem (AP) has been extensively used in manufacturing and developing service systems, to optimally resolve the problem of assigning N duties to N employees to optimize the total resources. Furthermore, in AP, N employees must be assigned N number of duties, where in each employee must complete their individual assigned duty. However, because of personal ability or other reasons, each employee may spend a different amount of resources to complete various duties. The objective is to assign each duty to the appropriate employee to optimize the total utilization of resources and to complete all duties. Two types of objectives are generally measured in AP: maximization and minimization. Minimization refers to minimizing aspects such as duty cost and total duration, whereas maximization refers to maximizing aspects such as the overall manufacturing profit and overall sale of manufacture.

Responsible parameters that are considered for determining the assignment plan in a real-world scenario should not be precise but should be exaggerated by indistinctness and imprecision represented by linguistic terms which expressed by the DM. In such a scenario, the AP is converted into a fuzzy assignment problem (FAP). The concept of fuzzy set theory was introduced by Zadeh (1965), which providesa highly effective method for handling imprecise data. In the decision-making real world problems, AP is more advantageous by fuzzy theory, subjective preference of DM. The fuzzy models of AP have been described in detail in several papers (Biswas and Pramanik 2011; Lin and Wen 2004; Lin et al. 2011; Li et al. 2012; Tanaka et al. 1984; Kagade and Bajaj 2009, 2010; Kumar and Gupta 2011; Liu and Gao 2009; Gupta and Mehlawat 2013; Mukherjee and Basu 2010; Feng and Yang 2006).

For handling objective functions and\or constraints with fuzzy coefficients and fuzzy information of real-world decision-making problems, possibilistic decision-making models play a vital role. Possibility distribution converts the fuzzy objectives and\or constraints into crisp objectives and\or constraints with respect to three scenarios, optimistic, mostlikely, and pessimistic. In addition, possibility distribution is used to maintain the uncertainty of the problem until the solution is obtained (Gupta and Mehlawat 2014). Several studies in literature have employed possibility distribution to solve fuzzy objective function and\or constraint-based optimization problems (Tanaka et al. 1984; Luhandjula 1987; Rommelfanger et al. 1989; Rommelfanger 1989; Lai and Hwang 1992).

There are several studies on FMOAP available in literature. To the best of our knowledge, Yang and Liu (2005) obtained a solution for a fuzzy multi-objective assignment problem (FMOAP) through the dependent-chance goal programming model by using the tabu search algorithm based on fuzzy simulation. Gupta and Mehlawat (2014) proposed a possibilistic programming approach for FMOAPs to obtain the most favorable, most likely, and least favorable scenarios by using the linear membership function. Li et al. (2012) proposed two solution models for FAP by combining agenetic algorithm (GA) and the APs to express an actual execution strategy. Tapkan et al. (2013) provided a direct approach to solving FMOAPs by using fuzzy ranking methods to rank the objective function values and to determine the feasibility of the constraints within the bees algorithm (metaheuristic search algorithm). Tailor and Dhodiya (2016a) developed a hybrid approach to solving FMOAPs by using a GA and exponential membership function. The Yager’s ranking method was proposed in Biswas and Pramanik (2011) to solve FMOAPs by transforming the MOAP into an equivalent single objective AP problem. Pramanik and Biswas (2012) developed a priority-based fuzzy goal programming method for generalized trapezoidal FMOAPs. Thorani and Shankar (2013) developed a linear programming model for FMOAPs by employing various linear and nonlinear functions with L–R fuzzy numbers by using Yager’s ranking method. Esmaieli et al. (2011) solved the fuzzy multi-job and multi-company employee AP with penalty by using a GA.

The aforementioned approaches present the solution for FMOAP by using various techniques, such as the possibilistic approach by using a linear membership function, Yager’s ranking method, priority-based fuzzy goal programming method, and bees algorithm, etc. However, in real-world problems, the decision parameters are affected by various imprecise and vague factors that cannot be precisely calculated. Moreover, the DM’s review of the estimates may be based on partial knowledge about the task itself, which may affect the decision of task allocations to a particular employee. Under such circumstances, the task allocation decision becomes a one ofchoice from a fuzzy set of subjective interpretations. Therefore, in this paper, we propose a GA-based hybrid approach to solving FMOAP by using a fuzzy exponential membership function in which the FMOAP is converted into a single objective nonlinear optimization problem with some realistic constraints, and it is considered as a “NP-hard” problem. GA is an appropriate technique to solve such “NP-hard” problems (Gupta and Mehlawat 2013; Papadimitriou and Steiglitz 1982). It is a well-known random search and global optimization method, considering the aspects of evolution and natural selection, and an appropriate method for solving large-scale nonlinear, discrete, and non-convex optimization problems because it searches for optimal solutions by simulating the natural evolution process (Eiben and Smith 2003; Holland 1992; Mendes et al. 2009; Gupta and Mehlawat 2013). GAs are highly efficient in the resolution of various NP-hard problems, including resource allocation.

## Fuzzy multi-objective assignment problem formulation

 The main characteristics and assumptions of the FMOAP are as follows: (1) Each duty is completed by only one employee, and if an employee accepts more than one duty, all the duties must be completed. (2) It is not compulsory to assigned any duty to some employees. (3) The number of employees who have been assigned duties must be specified to balance the amount of work between the employees. (4) In the decision-making method, each employee’s working ability is considered. We assume that each employee is assigned the number of duties in a certain range.

### Fuzzy multi-objective assignment model

To formulate the mathematical model of FMOAP, the indices, parameters and variables are used as per Gupta and Mehlawat (2013, 2014). (1) *Indices* j and i respectively defined index of duties and employee; (2) *Parameters* employees = duties = n; number employees assigned duties = s; maximum duties assigned to each employee = $l_{i}$; (3) *Decision variables* $x_{ij}$ is represent the whether the ith employee is assigned to jth duties or not.

$$\begin{aligned} x_{ij}=\left\{ \begin{array}{ll} 1;&{}\quad {\rm if}\,{\rm i}{{\rm th}}\, {\rm employee}\,{\rm is}\, {\rm assigned}\,{\rm to}\, {\rm j}{{\rm th}} \, {\rm duty} \\ {0};&{}\quad {\rm otherwise} .\end{array}\right. \end{aligned}$$

### Formulation of objective functions

After completion of all duties, the total cost, total consumed time and the total achieved a quality level are given as follows (Gupta and Mehlawat 2014):

$$\begin{aligned} \widetilde{{z_1}} = \sum \limits _{i = 1}^n {\sum \limits _{j = 1}^n {\widetilde{{c_{ij}}}{x_{ij}}}},\quad \widetilde{{z_2}} = \sum \limits _{i = 1}^n {\sum \limits _{j = 1}^n {\widetilde{{t_{ij}}}{x_{ij}}}},\quad \widetilde{{z_3}} = \sum \limits _{i = 1}^n {\sum \limits _{j = 1}^n {\widetilde{{q_{ij}}}{x_{ij}}}} \end{aligned}$$

In this problem, the quality of the linguistic variables are rated as “good,” “medium good,” “fair,” “medium poor,” and “poor,” which are represented as (0, 1, 3), (1, 3, 5), (3, 5, 7), (5, 7, 9), and (7, 9, 10), respectively. The five levels represent the quality of the completed duties, where “good” and “poor” levels denote the most efficient and least efficient, respectively, that is, a shift from “good” to “poor” indicates that the quality decreases whereas the related fuzzy values increase. To maintain uniformity of objective functions, quality objective functions must be minimized (Gupta and Mehlawat 2014).

### Model constraints

As per the mentioned description of FMOAP, the constraints are formulated as follows:

1 $$\sum _{i=1}^{n}\sum _{j=1}^{n}x_{ij}=n$$

2 $$\sum _{i=1}^{n}x_{ij}=1,\quad j=1, 2,\ldots, n$$

3 $$\sum _{j=1}^{n}x_{ij} \le l_{i};\quad i=1, 2,\ldots, n$$

4 $$\sum _{i=1}^{n}\min \left\{ 1,\sum _{j=1}^{n}x_{ij}\right\} \ge s$$

5 $$x_{ij} \in \left\{ 0, 1\right\} ,\quad i=1, 2,\ldots, n,\quad j=1, 2,\ldots, n.$$

### Decision problem

The fuzzy multi-objective assignment problem is now formulated as follows:

$$\begin{aligned}&{\mathbf{(Model}}{\text{-}}{{\bf 1)}} \\&\left( {\widetilde{{z_1}},\,\widetilde{{z_2}},\,\widetilde{{z_3}}} \right) = \left( {\sum \limits _{i = 1}^n {\sum \limits _{j = 1}^n {\widetilde{{c_{ij}}}{x_{ij}}}},\,\sum \limits _{i = 1}^n {\sum \limits _{j = 1}^n {\widetilde{{t_{ij}}}{x_{ij}}}},\,\sum \limits _{i = 1}^n {\sum \limits _{j = 1}^n {\widetilde{{q_{ij}}}{x_{ij}}}} } \right) \\&{\rm Subject}\,{\rm to}{:}\,(1){-}(5) \end{aligned}$$

## Some preliminaries

### Possibilistic programming approach

The collection data on real-world problems generally involve some type of uncertainty. In fact, many pieces of information cannot be quantified because of their nature and hence are represented using fuzzy numbers. These types of fuzzy numbers are modeled using possibility distribution (Hsu and Wang 2001; Buckley 1988; Gupta and Mehlawat 2014; Wang and Liang 2005; Lai and Hwang 1992). Possibilistic distribution has been used in many crucial applications to solve fuzzy optimization models with imprecise coefficients in the objective function. Thus, we converted the FMOAP model into an auxiliary crisp multi-objective optimization model by using the possibilistic approach (Gupta and Mehlawat 2014).

### Triangular possibilistic distribution (TPD)

 Because of the imprecise nature of the uncertain parameters, the triangular possibilistic distribution (TPD) is commonly used due to its simplicity and computational effectiveness in obtaining data.

**Fig. 1:**
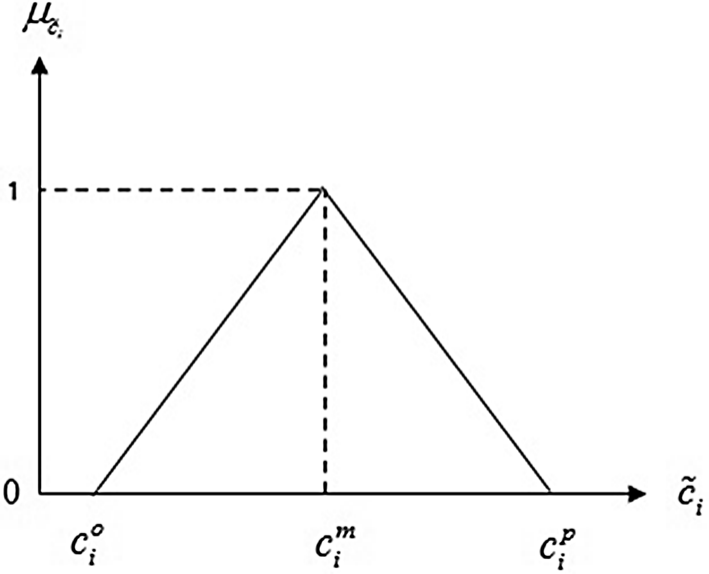
TPD of $$c_{i}$$

In realistic circumstances, a DM can construct the TPD by using ($c_{i}^{m}$), ($c_{i}^{o}$) and ($c_{i}^{p}$), most possible value (possibility degree = 1), the most optimistic value (possibility degree = 0) and the most pessimistic value (possibility degree = 0) respectively. According to Fig. 1, objective function cost is defined at three positions as $\left( {c_1^m, 1}\right) , \left( {c_1^p, 0} \right)$, and $\left( {c_1^o, 0}\right)$ which is minimized by shifting the three positions of TPD to the left because vertical coordinates of the points are fixed by 0 or 1 (Gupta and Mehlawat 2014). Thus, only the three horizontal coordinates are considered.

### *α*-Level sets

An *α*-level set is the most essential theory to establish an association between traditional and fuzzy set theories, which was introduced by Zadeh (1965). The *α*-level reflects the confidence of the DM regarding his fuzzy judgment; it can also be termed as the confidence level. The smallest *α*-value yields an interval judgment with a large spared, which indicates a high level of pessimism and uncertainty. The largest *α*-value yields a smaller but more optimistic judgment in which the upper and lower bounds have a greater degree of membership in the initial fuzzy sets. Several researchers (Tanaka et al. 1984; Luhandjula 1987; Rommelfanger et al. 1989; Rommelfanger 1989; Tailor and Dhodiya 2016a; Lai and Hwang 1992) have used this *α*-level set concept to find the solutions for fuzzy optimization-related problems; therefore, we also used this concept in the present study to determine the confidence of the DM with respect to his fuzzy judgment.

## Formulation of multi-objective 0–1 programming model

To convert the model-1 into auxiliary CMOP problem, we used TPD strategy to treat the imprecise objectives. The cost objective function is described as

6 $$\begin{aligned} \min \,\widetilde{{z_1}} &= \min \left( z_1^o, z_1^m, z_1^p\right) = \sum \limits _{i = 1}^n {\sum \limits _{j = 1}^n {\widetilde{{c_{ij}}}{x_{ij}}}} \\ &= \min \left( {\sum \limits _{i = 1}^n {\sum \limits _{j = 1}^n {c_{ij}^o{x_{ij}}}},\,\sum \limits _{i = 1}^n {\sum \limits _{j = 1}^n {c_{ij}^m{x_{ij}}}},\,\sum \limits _{i = 1}^n {\sum \limits _{j = 1}^n {c_{ij}^p{x_{ij}}}} } \right) \end{aligned}$$

where $c_{ij} =\left( c_{ij}^{o}, c_{ij}^{m},c_{ij}^{p} \right)$, which can be considered as follows:

7 $$\left( \min \, z_{11}, \min \, z_{12}, \min \, z_{13}\right) =\, \left( \sum _{i=1}^{n}\sum _{j=1}^{n}c_{ij}^{o} x_{ij}, \sum _{i=1}^{n}\sum _{j=1}^{n}c_{ij}^{m} x_{ij}, \sum _{i=1}^{n}\sum _{j=1}^{n}c_{ij}^{p} x_{ij} \right)$$

Equations (6) and (7) is associated with optimistic scenario, the most likely scenario and the pessimistic scenario respectively.

Using the *α*-level sets concepts ($0\le \alpha \le 1$), each $c_{ij}$ can be stated as $\left( c_{ij} \right) _{\alpha }=\left( \left( c_{ij} \right) _{\alpha }^{o},\left( c_{ij}\right) _{\alpha }^{m},\left( c_{ij} \right) _{\alpha }^{p} \right)$, where $\left( c_{ij} \right) _{\alpha }^{o} =c_{ij}^{o} +\alpha \left( c_{ij}^{m} -c_{ij}^{o} \right) , \left( c_{ij} \right) _{\alpha }^{m} =c_{ij}^{m}, \left( c_{ij}\right) _{\alpha }^{p} =c_{ij}^{p} -\alpha \left( c_{ij}^{p}-c_{ij}^{m} \right).$

Hence, Eq. (7) can be written as:

8 $$\left( \min \, z_{11}, \min \, z_{12}, \min \, z_{13} \right) =\, \left( \sum _{i=1}^{n}\sum _{j=1}^{n}\left( c_{ij}\right) _{\alpha }^{o} x_{ij}, \sum _{i=1}^{n}\sum _{j=1}^{n}\left( c_{ij} \right) _{\alpha }^{m} x_{ij}, \sum _{i=1}^{n}\sum _{j=1}^{n}\left( c_{ij} \right) _{\alpha }^{p} x_{ij}\right)$$

Similarly, multi-objective optimization problem (MOP) model of time and quality objective function are as follows:

9 $$\left( \min \, z_{21}, \min \, z_{22}, \min \, z_{23}\right) =\, \left( \sum _{i=1}^{n}\sum _{j=1}^{n}\left( t_{ij}\right) _{\alpha }^{o} x_{ij}, \sum _{i=1}^{n}\sum _{j=1}^{n}\left( t_{ij} \right) _{\alpha }^{m} x_{ij}, \sum _{i=1}^{n}\sum _{j=1}^{n}\left( t_{ij} \right) _{\alpha }^{p} x_{ij}\right)$$

10 $$\left( \min \, z_{31}, \min \, z_{32}, \min \, z_{33}\right) =\left( \sum _{i=1}^{n}\sum _{j=1}^{n}\left( q_{ij}\right) _{\alpha }^{o} x_{ij}, \sum _{i=1}^{n}\sum _{j=1}^{n}\left( q_{ij} \right) _{\alpha }^{m} x_{ij}, \sum _{i=1}^{n}\sum _{j=1}^{n}\left( q_{ij} \right) _{\alpha }^{p} x_{ij}\right)$$

### Auxiliary multi-objective 0–1 programming model

To determine the optimistic, most-likely, and pessimistic scenarios by using the *α*-level set concept, the FMOAP is converted into a crisp MOAP also called as an auxiliary multi-objective 0–1 programming model (Gupta and Mehlawat 2014; Tailor and Dhodiya 2016a), which is defined as follows:

11 $$\begin{aligned}&{\mathbf{(Model}}{\text{-}}{\textbf{2)}} \\&\left( {\min \,{z_{11}},\,\min \,{z_{12}},\,\min \,{z_{13}},\,\min \,{z_{21}},\,\min \,{z_{22}},\,\min \,{z_{23}},\,\min \,{z_{31}},\,\min \,{z_{32}},\,\min \,{z_{33}}} \right) \\&\quad =\left( \begin{array}{l} \sum \nolimits _{i = 1}^n {\sum \nolimits _{j = 1}^n {\left( {{c_{ij}}} \right) _\alpha ^o{x_{ij}}}},\,\sum \nolimits _{i = 1}^n {\sum \nolimits _{j = 1}^n {\left( {{c_{ij}}} \right) _\alpha ^m{x_{ij}}}},\,\sum \nolimits _{i = 1}^n {\sum \nolimits _{j = 1}^n {\left( {{c_{ij}}} \right) _\alpha ^p{x_{ij}}}},\\ \sum \nolimits _{i = 1}^n {\sum \nolimits _{j = 1}^n {\left( {{t_{ij}}} \right) _\alpha ^o{x_{ij}}}},\,\sum \nolimits _{i = 1}^n {\sum \nolimits _{j = 1}^n {\left( {{t_{ij}}} \right) _\alpha ^m{x_{ij}}}},\,\sum \nolimits _{i = 1}^n {\sum \nolimits _{j = 1}^n {\left( {{t_{ij}}} \right) _\alpha ^p{x_{ij}}}},\,\\ \sum \nolimits _{i = 1}^n {\sum \nolimits _{j = 1}^n {\left( {{q_{ij}}} \right) _\alpha ^o{x_{ij}}}},\sum \nolimits _{i = 1}^n {\sum \nolimits _{j = 1}^n {\left( {{q_{ij}}} \right) _\alpha ^m{x_{ij}}},\,\sum \nolimits _{i = 1}^n {\sum \nolimits _{j = 1}^n {\left( {{q_{ij}}} \right) _\alpha ^p{x_{ij}}}} } \, \end{array} \right) \end{aligned}$$

Under the constraints (1)–(5).

## Solution method for auxiliary model

To characterize the indistinct aspiration level of the DM, fuzzy membership functions such as linear, piecewise linear, exponential, and tangent are used. Out of these, the linear membership function is most commonly used because it is defined by two fixed points, the upper bound and lower bound of the objective, and also considered only a violent calculation of real-world circumstances. In addition, membership functions are used for describing the behavior of uncertain values, fuzzy data use, and preference etc. In such situation, the nonlinear membership function provides a more efficient representation than others to reflect the reality as the marginal rate of increasing membership values as a function of model parameter, which is not constant (Gupta and Mehlawat 2013).

GA is one of the most adaptive optimization search methodologies, which is based on natural genetics, natural selection, and survival of the fittest in a biological system. It mimics the evaluating principle and chromosome processing of natural genetics (Eiben and Smith 2003; Esmaieli et al. 2011; Gen et al. 1995; Holland 1992; Li et al. 1997; Mendes et al. 2009; Gupta and Mehlawat 2013; Sivanandam and Deepa 2007; Tailor and Dhodiya 2016a, b). To determine the solution of a single optimization FMAOP through GA, the chromosomes are first encoded according to the problem and a fitness function is defined for measuring the chromosomes. Subsequently, three operators, selection, crossover, and mutation, are applied to generate the new population. The selection process involves the formation of a parent population for creating the next generation. The crossover process involves the selection of two parent chromosomes to produce a new offspring chromosome. Mutation refers to randomly altering the selected positions in a selected chromosome (Gupta and Mehlawat 2013; Tailor and Dhodiya 2016b). Thus, the new population is generated by replacing some chromosomes in the parent population with those of the children population to determine effective solutions for FMOAPs (Tailor and Dhodiya 2016b).

This section presents a GA-based hybrid approach to determining the most efficient solution for an FMOAP by using the exponential membership function to characterize the indistinct aspiration levels of the DM. In addition, this approach provides greater flexibility to solve multi-objective optimization problems by considering the various choices of aspiration level for each objective function. This approach optimizes each objective by maximizing the degree of satisfaction with respect to cost, time, and quality to provide more effective assignment plans.

### Steps for find the solution of FMOAP using genetic algorithm based approach

The step-wise description of the proposed genetic algorithm based approach to finding the assignment plans of the FMOAP is as follows:

**Step-1**: Formulate the model-1 of FMOAP, using appropriate triangular possibilities distribution.
**Step-2**: According to confidence level *α*, define the crisp objective function model (model-2).
**Step-3**: Findout the positive ideal solution (PIS) and negative ideal solution (NIS) (Gupta and Mehlawat 2014) for each objective function of the model-2.
**Step-4**: Find fuzzy exponential membership value for $z_{ij} ({\rm i}=1, 2, 3; {\rm j}=1, 2, 3)$.
  12 $$\begin{aligned} \mu _{z_{ij}}^{E} \left( x\right) =\left\{ \begin{array}{ll} 1;&{}\quad {\rm if}\, \, z_{ij} \le z_{ij}^{{\rm PIS}} \\ \frac{e^{-S\psi _{ij} \left( x\right) } -e^{-S}}{1-e^{-S}};&{}\quad {\rm if}\, \, z_{ij}^{{\rm PIS}}<z_{ij} <z_{ij}^{{\rm NIS}}\\ 0;&{}\quad {\rm if}\, \, z_{ij} \ge z_{ij}^{{\rm NIS}} \end{array}\right. \end{aligned}$$
  where, $\psi _{ij} \left( x\right) =\frac{z_{ij} -z_{ij}^{{\rm PIS}} }{z_{ij}^{{\rm NIS}} -z_{ij}^{{\rm PIS}}}$ and S is non-zero shape parameter given by DM that $0\le \mu _{z_{ij}} \left( x\right) \, \le 1\,$. For $S>0\, (S<0)$, the membership function is strictly concave (convex) in [$z_{ij}^{{\rm PIS}}, z_{ij}^{{\rm NIS}}$]. The value of this fuzzy membership function allows us to model the grades of precision in corresponding objective function (Gupta and Mehlawat 2013).
**Step-5**: Fuzzy membership functions are comprehensive by using the product operator. Thus, FMOAP can be written in the single-objective optimizationproblem (SOP) as follows:
  $$\begin{aligned}&{\mathbf{(Model}}{\text{-}}{{\bf 3)}} \\&\max \, W = \prod \limits _{i = 1}^3 {\prod \limits _{j =1}^3 {{\mu _{{z_{ij}}}}}} \\&{\rm Subject}\,{\rm to}{:}\,{\rm Constraints}\,(1{-}5) \end{aligned}$$
  13 $${\mu _{{z_{ij}}}}\left( x \right) - \overline{{\mu _{{z_{ij}}}}} \left( x \right) \ge 0;\quad i = 1, 2, 3;\quad j = 1, 2, 3$$
  14 $$\mu _{z_{2j}} \left( x\right) -\overline{\mu _{z_{2j}} }\left( x\right) \ge 0;\quad j=1, 2, 3$$
  15 $$\mu _{z_{3j}} \left( x\right) -\overline{\mu _{z_{3j}} }\left( x\right) \ge 0;\quad j=1, 2, 3$$
  where $\overline{\mu }_{z_{ij}} \left( x\right) ; i=1, 2, 3; j=1, 2,\, 3\,$ is the desired aspiration level of fuzzy goals corresponding to each objective. The above model can be solved for varying aspiration levels of the DM regarding the achievement of various fuzzy membership functions (Gupta and Mehlawat 2013).
**Step-6**: To solve the single-objective optimization problem model-3 of the FMOAP, GA is used with various choices of the shape parameter.

- *Chromosome encoding*
  To generate a solution for the FMOAP, the data structure of chromosomes must be considered, which represents the solution to the problem in the encoding space. In the encoding space, we set all 0’s to all $n\times n$ genes on a chromosome, and then for a randomly selected gene on the chromosome, we set 1’s in each column exactly one and those in each row less or equal to $l_{i}$ that satisfies constraints (1)–(5) of model-3. Each component in the string (chromosome) can be uniquely expressed as $2^r$; where r is real value varying from 0 to ${\rm n}-1$.
- *Fitness function evaluation*
  In the GA, the fitness function is the major parameter for solving the FMOAP. The objective function of model-3 that satisfies constraints (1)–(5) and (13)–(15) is evaluated.
- *Selection*
  The selection operator is used to determine which chromosome from the current population will be used to reproduce a new child with higher fitness for the next population. It is carefully formulated to select the chromosome in the population with the highest fitness for mutation/next generation. This operator improves the average quality of the chromosomes in the population for the next generation by providing the chromosomes with the highest quality a higher chance to get copied into Gupta and Mehlawat (2013) and Tailor and Dhodiya (2016a, b).
  In this study, we used tournament selection for determining the solution for the FMOAP because of its efficiency and easy implementation. In tournament selection, N chromosomes are randomly selected from the population and compared with each other. The chromosome with the highest fitness (winner) is selected for the next generation and others are disqualified. This selection is continued until the number of winners is equal to the population size.
- *Crossover*
  After successful completion of tournament selection, the crossover operator is used to produce a new offspring for the next generation. The principle underlying crossover is that the offspring may exhibit a higher level of fitness than both parents if it inherits high-quality characteristics from each parent.
  To generate a solution for the FMOAP, we used the two-point crossover operator to generate a new offspring. In a two-point crossover, the gene values are exchanged between two random crossover points on the two selected parent chromosomes to generate the new offspring (Sivanandam and Deepa 2007; Tailor and Dhodiya 2016a).
- *Threshold construction*
  To maintain the population diversity after crossover, a threshold is constructed to generate the FMOAP’s solution. In this step, from the set of parenthood and childhood population some are selected for the new iteration.
  For constructing the threshold, one method of selecting the population may be to sort the entire population in an ascending order of their objective function values and selecting predetermined individual strings from each category. The population is divided into four categories on the basis of their objective function values: values above $\mu +3*\sigma$, values between $\mu +3*\sigma$ and $\mu$, values between $\mu$ and $\mu -3*\sigma$, and values less then $\mu -3*\sigma$. Thus, the most efficient string cannot be missed (Sivanandam and Deepa 2007; Tailor and Dhodiya 2016a, b).
- *Mutation*
  For recovering the lost genetic materials and for randomly disturbing genetic information, the mutation operator is applied. In this study, we applied the swap mutation (Gupta and Mehlawat 2013; Tailor and Dhodiya 2016a, b) out of the numerous mutation operators available. In a swap mutation, two random spots are selected in a string and the corresponding values are swapped between the positions.
  If we swap the string $\langle 1, 2, 3, 4, 5\rangle$ at second and fourth position then the new mutated string become $\langle 1, 4, 3, 2, 5\rangle$.
- *Termination criteria*
  When the algorithm completes a given number of iterations, it stops and provides the optimal solution as the output. The iteration process is repeated until a termination condition is reached.

After developing the algorithm, two cases were implemented: one with mutation and another without mutation. In both cases, the answer converged to the efficient solution for FMOAP (Tailor and Dhodiya 2016a, b).

If the obtained solution is accepted by the DM, then it is considered as the ideal compromise solution and the iteration is stopped, else the value ofis changed and steps 2–5 are repeated untila satisfactory solution is achieved.

### Algorithm

Algorithm of solution procedure for FMOAP is given as follows: 
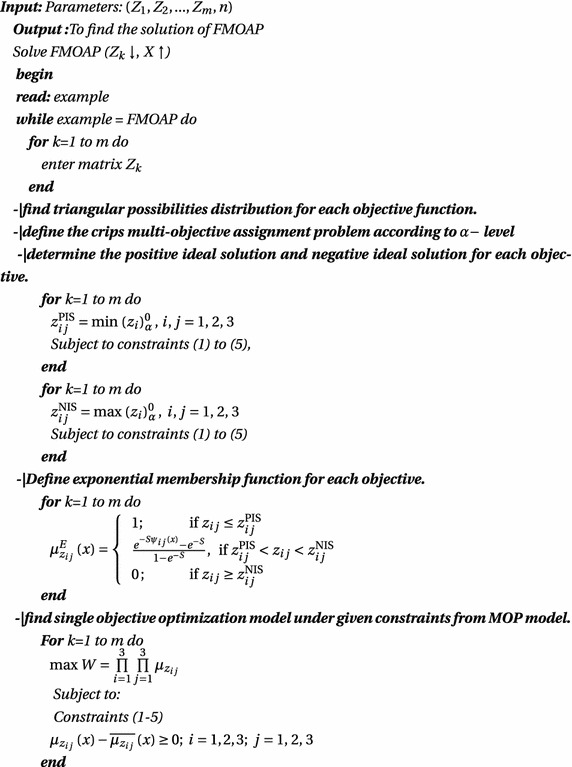
 
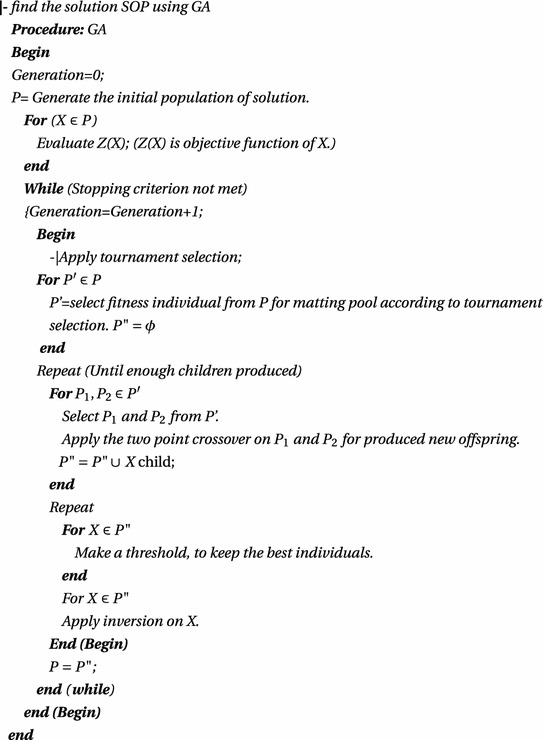


### Flowchart

**Fig. 2:**
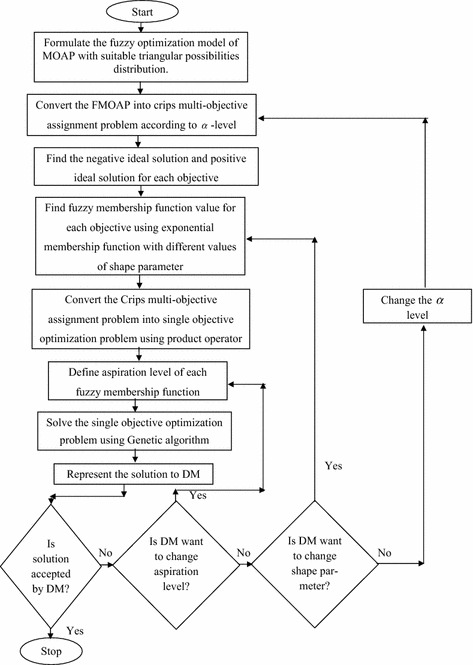
flowchart of the solution procedure of FMAOP

Figure 2 shows the flowchart of the solution procedure of FMAOP.

### Convergence criteria

A GA usually converges when no significant improvement is observed in the fitness values of the population from one generation to the next. GA converging at a global optima for an NP-hard problem is impossible, unless the optimum solution for a test data set is already known. In GA, the convergence criteria also depends on the problem size. In this study, we considered a problem with size $6 \times 6$ size problem. For this problem, we set the experimental parameters as follows: size = 4500, iterations = 90 at different values $\alpha =0.1, \alpha =0.5$ and $\alpha =0.9$. The experiment is presented in the following section.

## Numerical illustration

To justify proposed method, numerical illustration of FMOAP has been referred from the article of the Gupta and Mehlawat (2014) which shown in Table 1. To evaluate fuzzy cost-time-quality objective assignment problem, the model is coded. It is solved by Matlab and all tests are carried out on an Intel (R)-core i5 CPU@ 2.60 GHz computer with 4 GB of RAM. The primary attributes for solving the problems summarized as follows: Number of workers = Number of jobs = 6, $l_{i} = 2$, s = 4, population size = 4500, iterations = 100.

**Table 1:** Cost-time-quality matrix

Worker (i)	Job (j)					
	Job-1	Job-2	Job-3	Job-4	Job-5	Job-6
*Worker-1*						
$c_{ij}$	(4, 6, 8)	(3, 4, 6)	(4, 5, 8)	(6, 8, 11)	(7, 10, 14)	(4, 6, 7)
$t_{ij}$	(2, 4, 5)	(16, 20, 24)	(7, 9, 12)	(2, 3, 5)	(5, 8, 10)	(7, 9, 12)
$q_{ij}$	(0, 1, 3)	(1, 3, 5)	(0, 1, 3)	(0, 1, 3)	(0, 1, 3)	(3, 5, 7)
*Worker-2*						
$c_{ij}$	(4, 6, 7)	(4, 5, 7)	(5, 6, 9)	(3, 5, 7)	(6, 9, 11)	(6, 8, 11)
$t_{ij}$	(4, 6, 9)	(15, 18, 22)	(6, 8, 12)	(5, 7, 10)	(14, 17, 20)	(6, 8, 10)
$q_{ij}$	(1, 3, 5)	(3, 5, 7)	(1, 3, 5)	(3, 5, 7)	(5, 7, 9)	(3, 5, 7)
*Worker-3*						
$c_{ij}$	(8, 11, 14)	(5, 7, 9)	(2, 4, 6)	(5, 8, 12)	(2, 3, 4)	(3, 4, 6)
$t_{ij}$	(2, 3, 4)	(6, 8, 10)	(17, 20, 24)	(5, 7, 10)	(12, 15, 18)	(5, 7, 10)
$q_{ij}$	(0, 1, 3)	(5, 7, 9)	(3, 5, 7)	(1, 3, 5)	(3, 5, 7)	(5, 7, 9)
*Worker-4*						
$c_{ij}$	(7, 9, 12)	(7, 10, 12)	(6, 8, 11)	(4, 6, 8)	(8, 10, 12)	(3, 4, 6)
$t_{ij}$	(10, 12, 16)	(10, 13, 16)	(12, 14, 18)	(4, 6, 9)	(7, 9, 12)	(8, 10, 14)
$q_{ij}$	(3, 5, 7)	(7, 9, 10)	(1, 3, 5)	(3, 5, 7)	(1, 3, 5)	(1, 3, 5)
*Worker-5*						
$c_{ij}$	(3, 4, 6)	(4, 6, 8)	(5, 7, 10)	(7, 9, 12)	(6, 8, 12)	(5, 7, 10)
$t_{ij}$	(7, 9, 12)	(5, 8, 11)	(5, 7, 10)	(11, 14, 18)	(3, 5, 8)	(7, 9, 12)
$q_{ij}$	(1, 3, 5)	(7, 9, 10)	(5, 7, 9)	(3, 5, 7)	(1, 3, 5)	(1, 3, 5)
*Worker-6*						
$c_{ij}$	(2, 3, 4)	(4, 5, 7)	(8, 11, 15)	(8, 10, 13)	(9, 12, 15)	(6, 8, 12)
$t_{ij}$	(14, 17, 21)	(10, 13, 16)	(2, 3, 5)	(3, 5, 8)	(10, 13, 17)	(5, 7, 10)
$q_{ij}$	(1, 3, 5)	(1, 3, 5)	(3, 5, 7)	(5, 7, 9)	(3, 5, 7)	(5, 7, 9)

Table 2 gives the PIS and NIS for each objective functions for $\alpha =0.1, \alpha =0.5$ and $\alpha =0.9$. These values are used to define the exponential membership function. The corresponding values are obtained in below table.

**Table 2:** PIS and NIS for fuzzy objective functions

*α*-Level	Solutions	Objectives								
		$z_{11}$	$z_{12}$	$z_{13}$	$z_{21}$	$z_{22}$	$z_{23}$	$z_{31}$	$z_{32}$	$z_{33}$
$\alpha =0.1$	PIS	15.8	23	32	20	29	40.7	3.9	12	22.8
	NIS	46.6	61	77.2	81.8	98	118.7	31.2	42	51.9
$\alpha =0.5$	PIS	19	23	28	24	29	35.5	7.5	12	18
	NIS	53	61	70	89	98	109.5	36	42	47.5
$\alpha =0.9$	PIS	22.2	23	24	28	29	30.3	11.1	12	13.2
	NIS	59.4	61	62.8	96.2	98	100.3	40.8	42	43.1

According to triangular possibility distribution, the assignment plans for FMOAP are reported in below tables with different values of the shape parameters and aspiration levels which specified by the DM. We use here different values of for $\alpha =0.1,\alpha =0.5$ and $\alpha =0.9$ to reflect the different scenario of DM’s confidence about fuzzy decision.

We have stated the results by taking different estimation of the aspiration levels for each combination of the shape parameters shown in Table 3.

**Table 3:** Different values of shape parameters and aspiration level

Case	Shape parameter $\left( K_{1}, K_{2}, K_{3} \right)$	Aspiration level $\left( \bar{\mu }_{Z_{1j}} (x),\bar{\mu }_{Z_{2j}} (x), \bar{\mu }_{Z_{3j}} (x)\right)$
Case-1	(−5, −1, −2)	0.7, 0.8, 0.9
Case-2	(−5, −1, −2)	0.8, 0.85, 0.7
Case-3	(−5, −1, −2)	0.9, 0.7, 0.8
Case-4	(−1, −2, −5)	0.7, 0.8, 0.85
Case-5	(−1, −2, −5)	0.8, 0.7, 0.75
Case-6	(−2, −5, −1)	0.8, 0.85, 0.7
Case-7	(−2, −5, −1)	0.9, 0.75, 0.8

Table 4 shows the assignment plans for each objective at different values of confidence level $\alpha = 0.1, 0.5$ and 0.9 with different values of the shape parameters and different estimates of aspiration levels. From the above table, we also show that change in confidence level influence spreads of the objective function i.e. as the confidence level is increasing, the influence of uncertainty in the fuzzy preference of the DM decreases.

**Table 4:** Summary results for $\alpha =0.1, \alpha =0.5$ and $\alpha =0.9$

*α*	Case	*W*	Membership values $\left( \mu _{Z_{1j}}, \mu _{Z_{2j}}, \mu _{Z_{3j}} \right)$	Objective values $Z_{1}, Z_{2}, Z_{3}$	Optimum allocations
$\alpha =0.1$	1	0.8954	(0.9111, 0.9241, 0.8954)	(32.1, 42, 57.3)	$x_{11}, x_{14}, x_{46}, x_{55}, x_{62}, x_{63}$
			(0.9185, 0.9189, 0.9136)	(28.1, 38, 51.5)	
			(0.9601, 0.9522, 0.9505)	(7, 16, 26.8)	
	2	0.8527	(0.9499, 0.9640, 0.9488)	(28.9, 37, 51.4)	$x_{11}, x_{14}, x_{23}, x_{46}, x_{55}, x_{62}$
			(0.8730, 0.8691, 0.8527)	(32.2, 43, 58.3)	
			(0.9869, 0.9777, 0.9769)	(5, 14, 24.8)	
	3	0.8611	(0.9127, 0.9343, 0.9070)	(32, 41, 56.3)	$x_{13}, x_{14}, x_{31}, x_{46}, x_{55}, x_{62}$
			(0.8626, 0.8691, 0.8611)	(33.1, 43, 57.4)	
			(1, 1, 1)	(3.9, 12, 22.8)	
	4	0.9115	(0.9115, 0.9419, 0.9265)	(22.7, 29, 40.7)	$x_{13}, x_{14}, x_{35}, x_{46}, x_{51}, x_{62}$
			(0.9450, 0.9471, 0.9449)	(47.3, 59, 75.2)	
			(0.9300, 0.9170, 0.9142)	(7, 16, 26.8)	
	5	0.8667	(0.9254, 0.9419, 0.9172)	(21.8, 29, 41.6)	$x_{11}, x_{34}, x_{35}, x_{46}, x_{51}, x_{62}$
			(0.9274, 0.9271, 0.9220)	(50.4, 63, 80.1)	
			(0.9032, 0.8711, 0.8667)	(8.1, 18, 28.8)	
	6	0.7799	(0.8000, 0.8248, 0.7799)	(24.9, 33, 46.5)	$x_{11}, x_{13}, x_{24}, x_{46}, x_{55}, x_{62}$
			(0.8913, 0.8850, 0.8771)	(36.3, 48, 63.3)	
			(0.9948, 0.9936, 0.9933)	(7, 16, 26.8)	
	7	0.8240	(0.8974, 0.9182, 0.9055)	(20.8, 28, 38.8)	$x_{11}, x_{13}, x_{24}, x_{35}, x_{36}, x_{62}$
			(0.8334, 0.8240, 0.8298)	(42.4, 55, 69.4)	
			(0.9709, 0.9709, 0.9690)	(13, 22, 32.8)	
$\alpha =0.5$	1	0.9080	(0.9178, 0.9241, 0.9080)	(36.5, 42, 50.5)	$x_{11}, x_{14}, x_{46}, x_{55}, x_{62}, x_{63}$
			(0.9178, 0.9189, 0.9158)	(32.5, 38, 45.5)	
			(0.9564, 0.9522, 0.9512)	(11, 16, 22)	
	2	0.8595	(0.9574, 0.9640, 0.9555)	(32.5, 37, 45)	$x_{11}, x_{14}, x_{23}, x_{46}, x_{55}, x_{62}$
			(0.8711, 0.8691, 0.8595)	(37, 43, 51.5)	
			(0.9826, 0.9777, 0.9773)	(9, 14, 20)	
	3	0.8644	(0.9241, 0.9343, 0.9191)	(36, 41, 49.5)	$x_{13}, x_{14}, x_{31}, x_{46}, x_{55}, x_{62}$
			(0.8657, 0.8691, 0.8644)	(37.5, 43, 51)	
			(1, 1, 1)	(7.5, 12, 18)	
	4	0.9155	(0.9271, 0.9419, 0.9328)	(25.5, 29, 35)	$x_{13}, x_{14}, x_{35}, x_{46}, x_{51}, x_{62}$
			(0.9460, 0.9471, 0.9458)	(52.5, 59, 68)	
			(0.9240, 0.9170, 0.9155)	(11, 16, 22)	
	5	0.8687	(0.9388, 0.9419, 0.9274)	(25, 29, 36)	$x_{13}, x_{34}, x_{35}, x_{46}, x_{51}, x_{62}$
			(0.9273, 0.9271, 0.9241)	(56, 63, 72.5)	
			(0.8884, 0.8711, 0.8687)	(12.5, 18, 24)	
	6	0.7983	(0.8124, 0.8248, 0.7983)	(28.5, 33, 40.5)	$x_{11}, x_{13}, x_{24}, x_{36}, x_{55}, x_{62}$
			(0.9120, 0.9076, 0.9053)	(38.5, 45, 53)	
			(0.9815, 0.9810, 0.9805)	(15, 20, 26)	
	7	0.8385	(0.8877, 0.9005, 0.8945)	(25, 29, 35)	$x_{11}, x_{13}, x_{35}, x_{36}, x_{44}, x_{62}$
			(0.8389, 0.8335, 0.8362)	(47, 54, 62)	
			(0.9709, 0.9709, 0.9698)	(17, 22, 28)	
$\alpha =0.9$	1	0.9183	(0.9236, 0.9241, 0.9209)	(40.9, 42, 43.7)	$x_{11}, x_{14}, x_{46}, x_{55}, x_{62}, x_{65}$
			(0.9189, 0.9189, 0.9183)	(36.9, 38, 39.5)	
			(0.9530, 0.9522, 0.9520)	(15, 16, 17.2)	
	2	0.8671	(0.9628, 0.9640, 0.9623)	(36.1, 37, 38.6)	$x_{11}, x_{14}, x_{23}, x_{46}, x_{55}, x_{62}$
			(0.8695, 0.8691, 0.8671)	(41.8, 43, 44.7)	
			(0.9786, 0.9777, 0.9776)	(13, 14, 15.2)	
	3	0.8681	(0.9326, 0.9343, 0.9313)	(40, 41, 42.7)	$x_{13}, x_{14}, x_{31}, x_{46}, x_{55}, x_{62}$
			(0.8684, 0.8691, 0.8681)	(41.9, 43, 44.6)	
			(1, 1, 1)	(11.1, 12, 13.2)	
	4	0.8719	(0.8738, 0.8773, 0.8719)	(33.2, 34, 35.6)	$x_{13}, x_{14}, x_{46}, x_{51}, x_{55}, x_{62}$
			(0.9778, 0.9779, 0.9774)	(47.8, 49, 50.8)	
			(0.9616, 0.9599, 0.9774)	(13, 14, 15.2)	
	5	0.8707	(0.9159, 0.9181, 0.9139)	(30.2, 31, 32.5)	$x_{13}, x_{24}, x_{46}, x_{51}, x_{55}, x_{62}$
			(0.9682, 0.9682, 0.9675)	(51.7, 53, 54.9)	
			(0.8754, 0.8711, 0.8707)	(16.9, 18, 19.2)	
	6	0.8191	(0.8226, 0.8248, 0.8191)	(32.1, 33, 34.5)	$x_{11}, x_{13}, x_{24}, x_{36}, x_{55}, x_{62}$
			(0.9085, 0.9076, 0.9072)	(43.7, 45, 46.6)	
			(0.9811, 0.9810, 0.9809)	(19, 20, 21.2)	
	7	0.8335	(0.8981, 0.9005, 0.8992)	(28.2, 29, 30.2)	$x_{11}, x_{13}, x_{35}, x_{36}, x_{44}, x_{62}$
			(0.8345, 0.8335, 0.8340)	(52.6, 54, 55.6)	
			(0.9709, 0.9709, 0.9707)	(21, 22, 23.2)	

### The convergence rate of GA for FMOAP

**Fig. 3:**
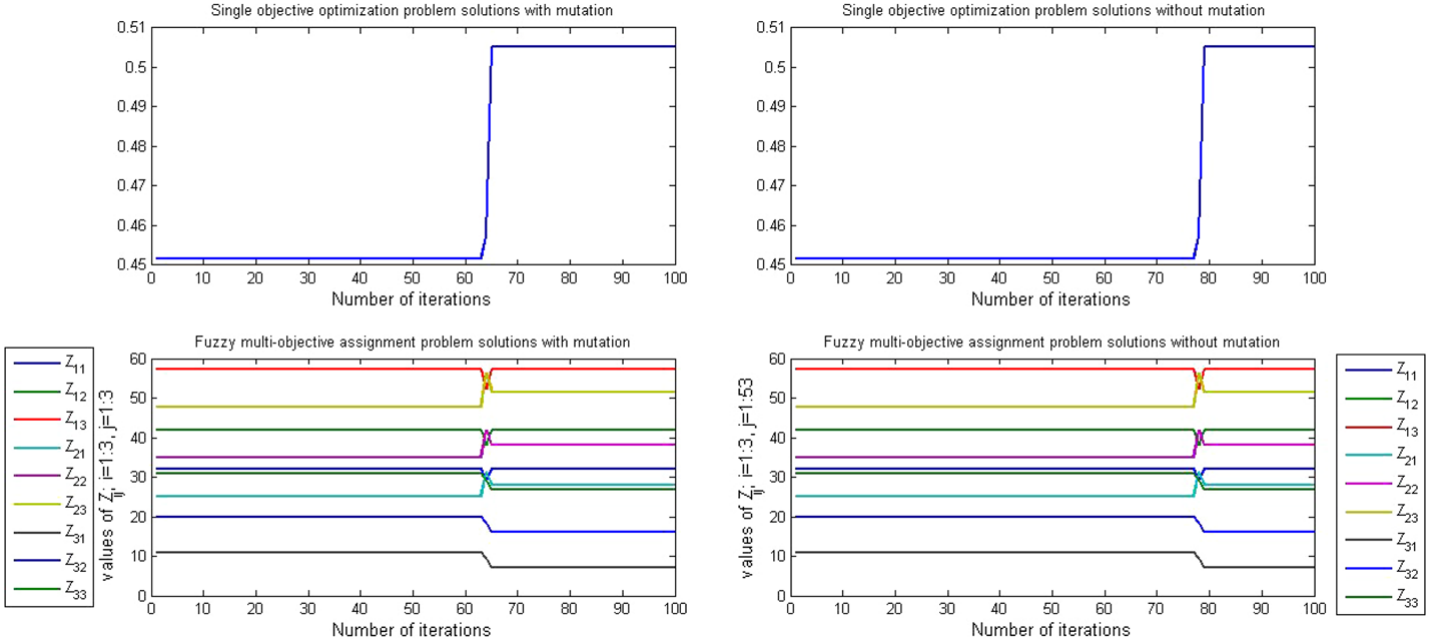
Convergence to the global optimization for SOP and FMOAP at $\alpha =0.1$ with ($-5, -1, -2$) shape parameter and (0.8, 0.85, 0.7) aspiration level in case of mutation and without mutation

**Fig. 4:**
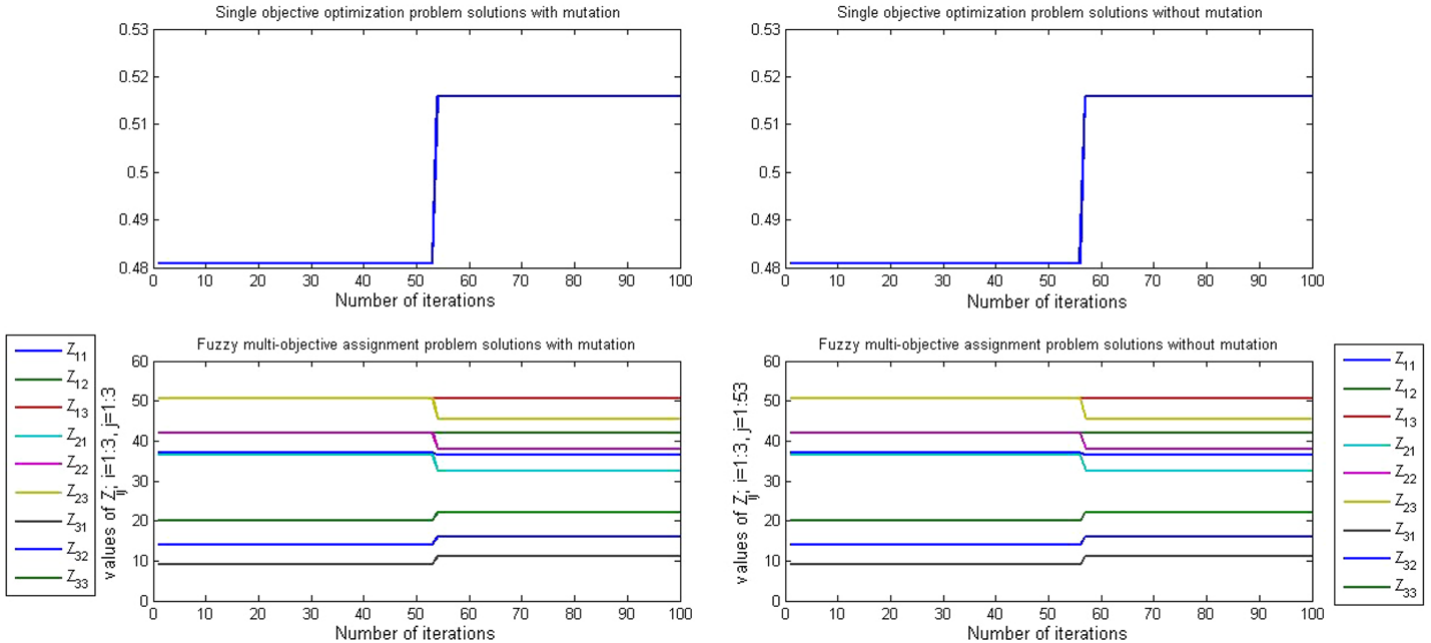
Convergence to the global optimization for SOP and FMOAP at $\alpha =0.5$ with ($-5, -1, -2$) shape parameter and (0.8, 0.85, 0.7) aspiration level in case of mutation and without mutation

**Fig. 5:**
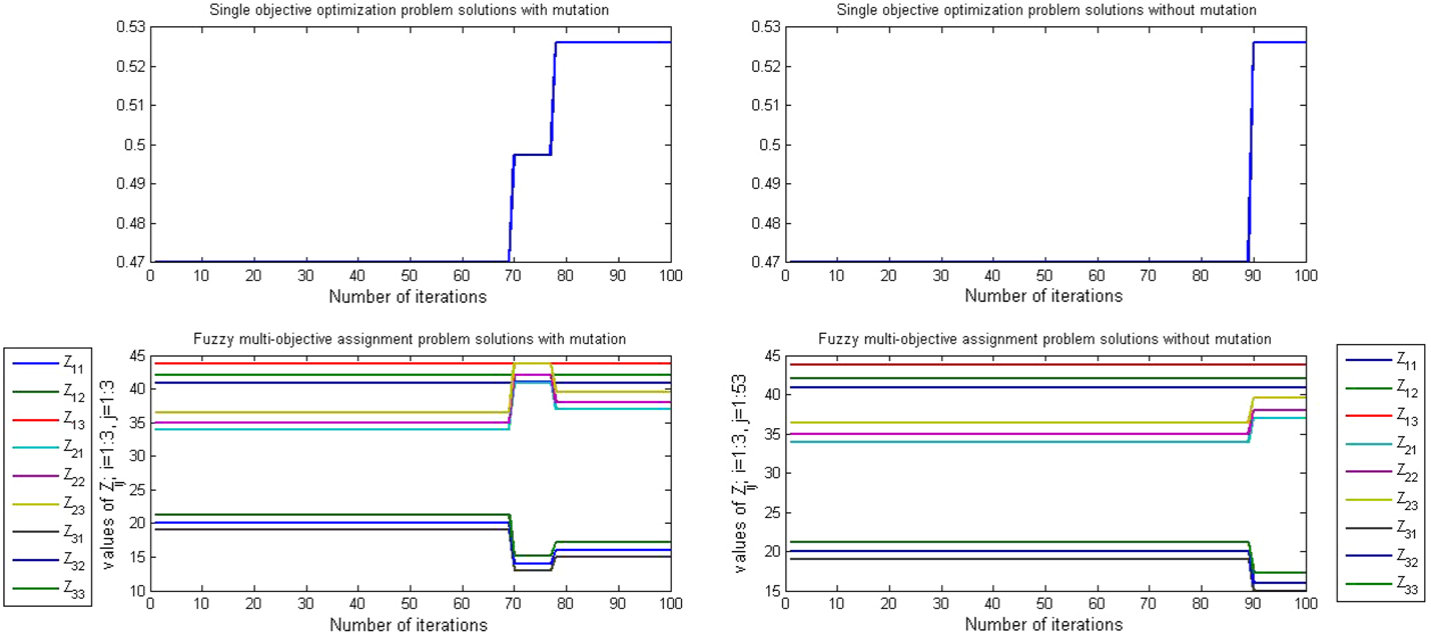
Convergence to the global optimization for SOP and FMOAP at $\alpha =0.9$ with ($-5, -1, -2$) shape parameter and (0.8, 0.85, 0.7) aspiration level in case of mutation and without mutation

Figures 3, 4 and 5 show that the efficient solution of SOP and FMOAP in case of using mutation operator and without using the mutation operator with ($-5, -1, -2$) shape parameter and (0.8, 0.85, 0.7) aspiration level respectively at $\alpha =0.1, \alpha = 0.5$ and $\alpha =0.9$. In with mutation case solution of SOP and FMOAP is converging after 65, 54, 78 iterations respectively, while in without mutation case solution of SOP and FMOAP is converging after 80, 59, 90 iterations respectively. Figures 3, 4 and 5 are also provided other alternative solution to DM as per their requirement.

**Fig. 6:**
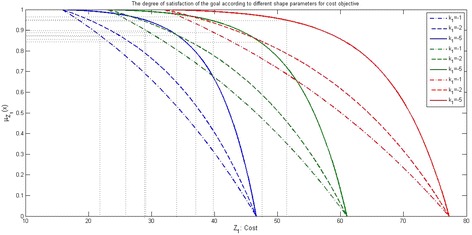
The degree of satisfaction of the goal according to different shape parameter for cost objective

**Fig. 7:**
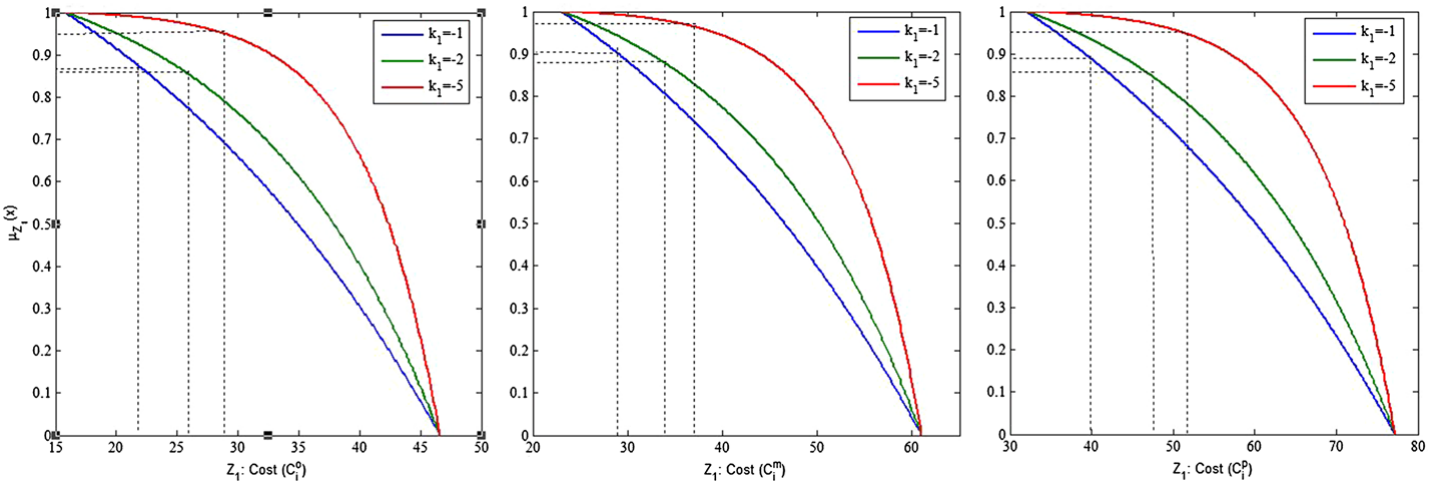
The degree of satisfaction of the goal of the cost objective for $\alpha =0.1$

**Fig. 8:**
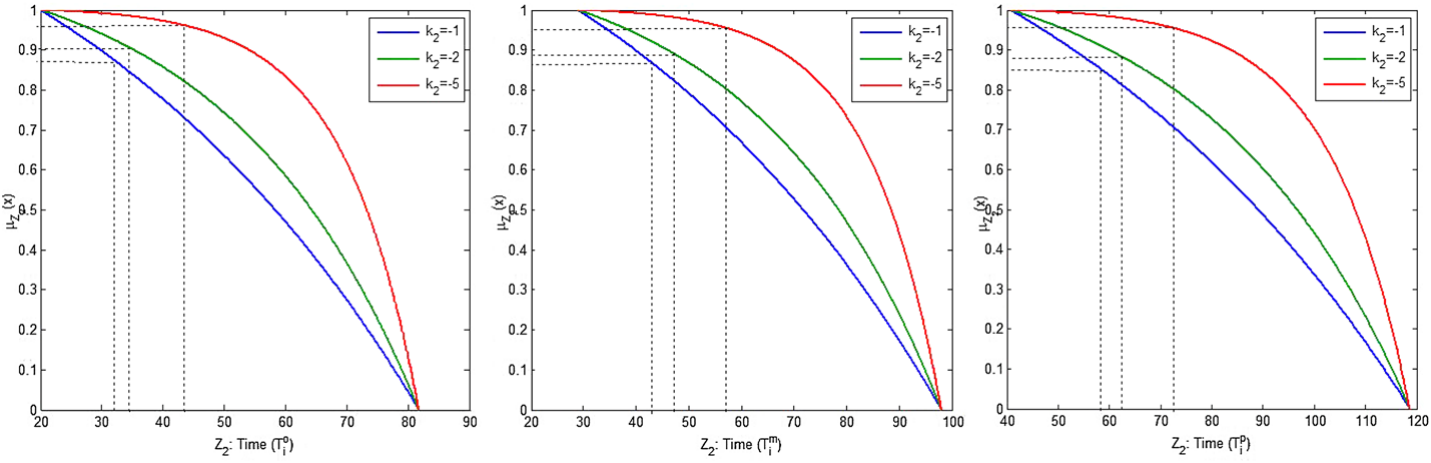
The degree of satisfaction of the goal of the time objective for $\alpha =0.1$

**Fig. 9:**
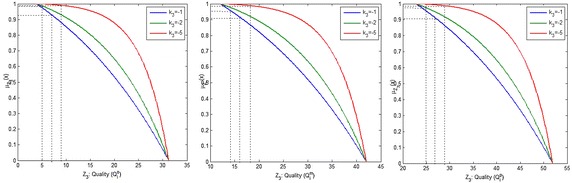
The degree of satisfaction of the goal of the quality objective for $\alpha =0.1$

Figures 6, 7, 8 and 9 show the variation of the goals (cost, time and quality objective) corresponding to different preference of shape parameters for $\alpha =0.1$. From these figures, it is clear that obtained solution having more influence of optimism then pessimism, representing possibility distribution corresponding to each objective function respectively.

**Fig. 10:**
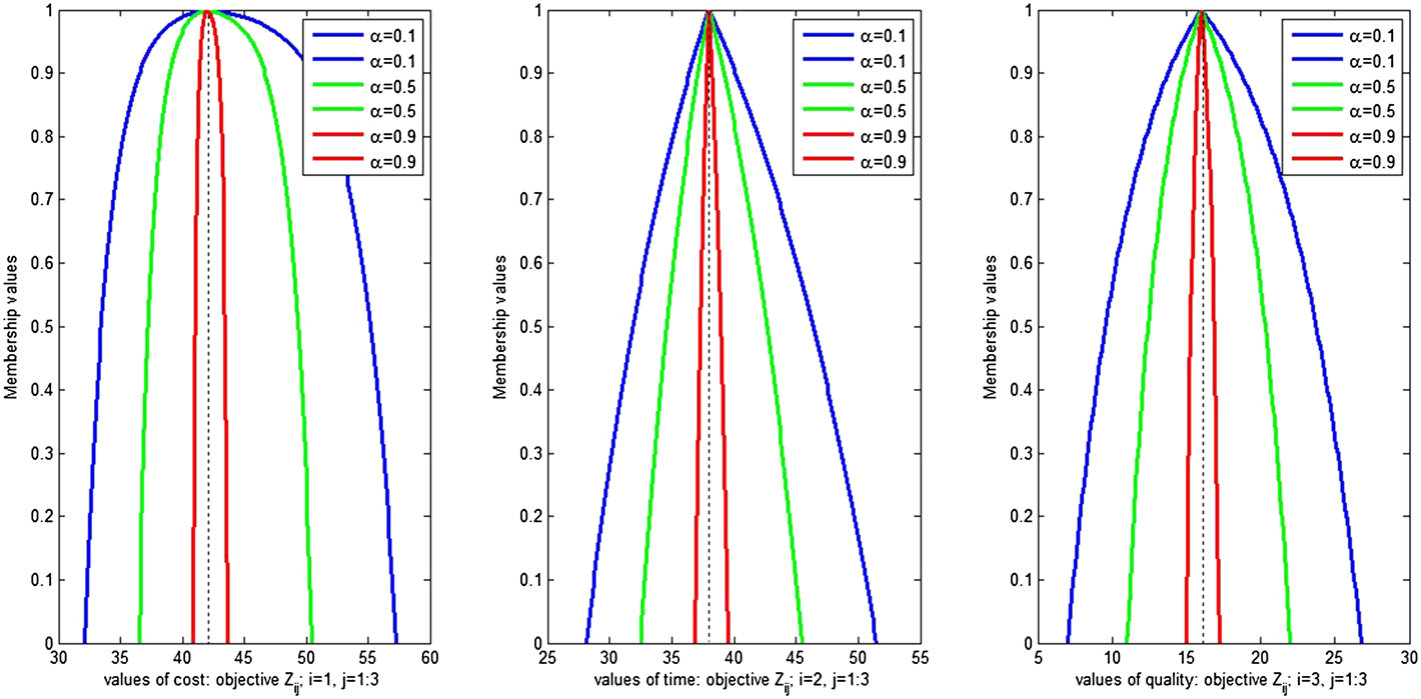
Possibilities distribution for cost, time and quality objectives with ($-5,-1,-2$) shape parameter and (0.8, 0.85, 0.7) aspiration level

**Fig. 11:**
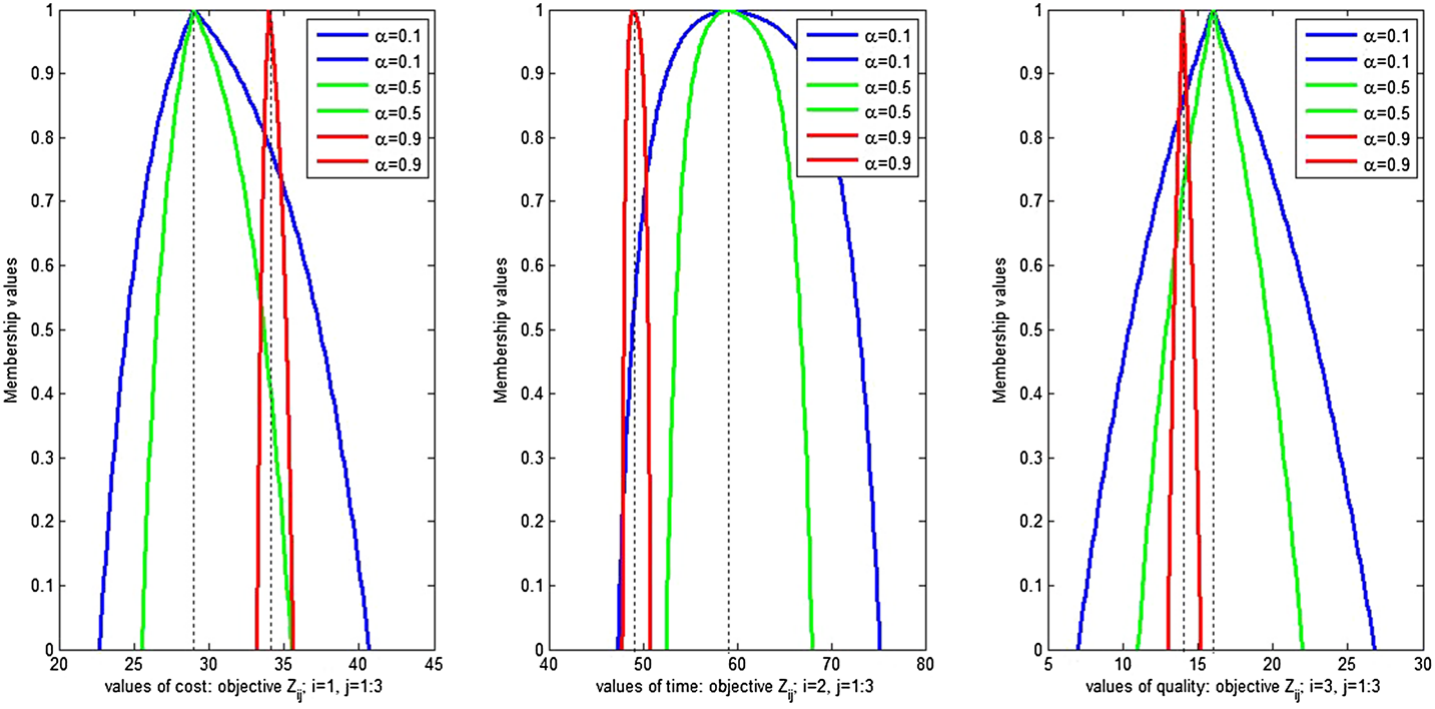
Possibilities distribution for cost, time and quality objectives with ($-2, -5, -1$) shape parameter and (0.8, 0.85, 0.7) aspiration level

**Fig. 12:**
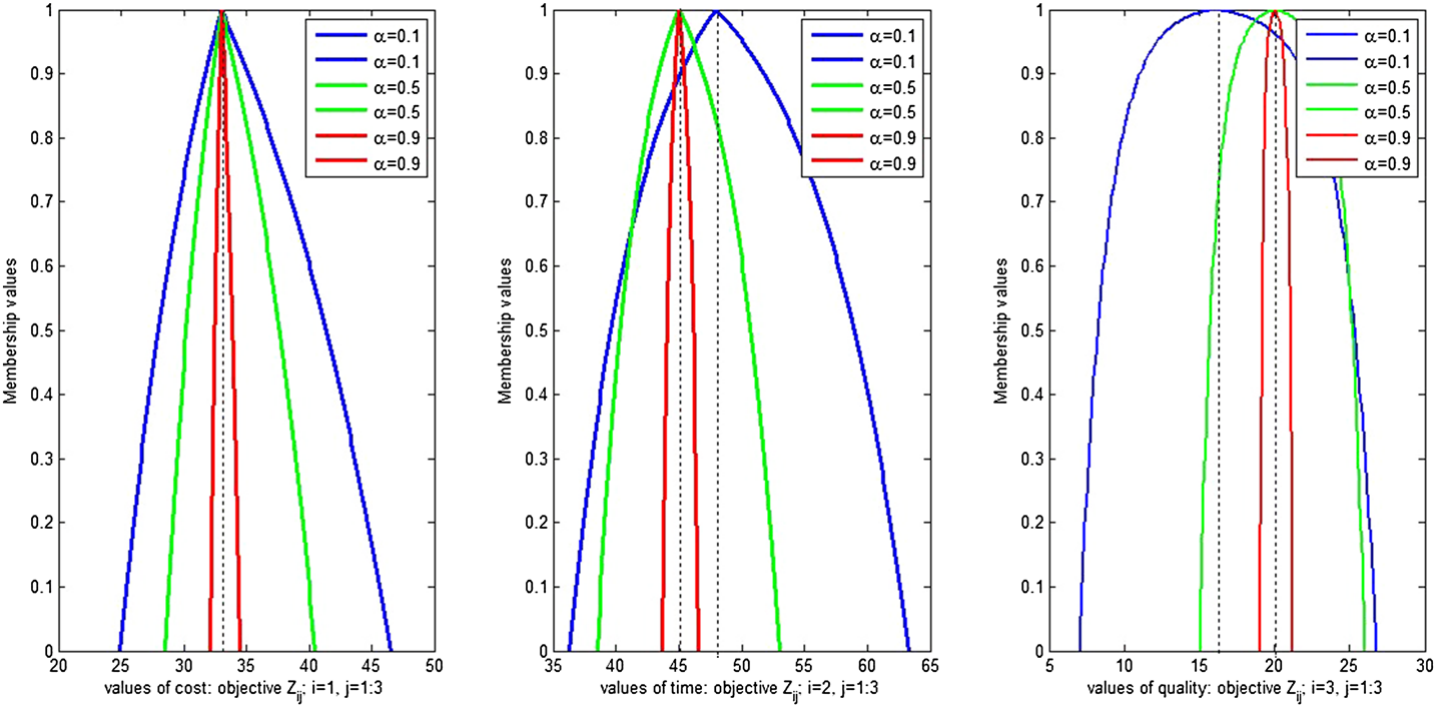
Possibilities distribution for cost, time and quality objectives with ($-1,-2,-5$) shape parameter and (0.7, 0.8, 0.85) aspiration level

Figures 10, 11 and 12 show the assignment plan for each objective at different confidence level $\alpha =0.1, 0.5$ and 0.9 for different shape parameter and different aspiration level. From these figures, we conclude that with the increase in *α*, the influence of uncertainty decreases in the DM fuzzy judgment. Moreover, the advantage of using the exponential membership function with various shape parameters for the FMOAP is presented. If the DM is not satisfied with an assignment plan, more plans can be generated by changing the values of the confidence level and shape parameters in the exponential membership functions, which allow us to investigate various fuzzy values of the DM.

To defuzzify the fuzzy number, Lai and Hwang (1992) provided the concept of most likely values to verify the efficiency of outputs. They determined crisp values for each objective corresponding to the triangular number. If cost $\tilde{C}=\left( C^{o}, C^{m}, C^{p} \right)$ is a triangular fuzzy number, then the crisp value of cost objective is given as $\tilde{C}=\left( \frac{C^{o} +4C^{m} +C^{p}}{6} \right)$ which provided the most likely value of the objective function.

Furthermore, if the DM is not satisfied with the obtained compromise solution, then the desired objective function can be improved as per the preference of the DM. For example, in an AP with fuzzy cost, time, and quality objectives, if the DM prioritizes the cost objective in determining the period of allocation plan, the solution that satisfies the cost objective function most favorably than others is selected by the DM. However, this can result in poor degrees of satisfaction level because the performance of one objective may be compensated by the efficient performance of others. Hence, the DM can select different solutions in different situations, according to his/her requirements. Therefore, to generate a new membership function, the upper bound of the selected objective function is modified using the DM’s preference. The model is resolved using new parameters and the iterations are continued until the DM terminates the process (Gupta and Mehlawat 2013, 2014; Tailor and Dhodiya 2016a).

 Tables 5, 6 and 7 are reported the preferred compromise solution which obtained by changing the upper bounds of various objectives with different values of confidence level, different values of shape parameters and different estimation of aspiration levels for different confidence level *α*.

**Table 5:** Compromised solutions with respect to improvement desired in various objective at confidence level $\alpha =0.1$ with different shape parameter

Case	Obj. function	Bounds	$\lambda$	Objective values	Solution variables
				($Z_{1}, Z_{2}, Z_{3}$)	$x_{ij}$
*Shape parameter: (−5, −1, −2)*					
Aspiration level: (0.8, 0.85, 0.7)					
1	Cost	$15.8 \le z_{11} \le 32,$	0.8025	(26.9, 35, 48.5),	$x_{11}, x_{23}, x_{36}, x_{44}, x_{55}$,$x_{62}$
		$23 \le z_{12} \le 41,$		(31.3, 43, 58.3),	
		$32 \le z_{13}\le 56.3$		(12.1, 22, 32.8)	
2	Quality	$3.9\le z_{31}\le 7,$	0.8611	(32, 41, 56.3),	$x_{13}, x_{14}, x_{31}, x_{46}, x_{55}$,$x_{62}$
		$12 \le z_{32} \le 16,$		(33.1, 43, 57.4),	
		$$22.8 \le z_{33} \le 26.8$$		(3.9, 12, 22)	
Aspiration level: (0.9, 0.7, 0.8)					
1	Cost	$15.8 \le z_{11}\le 28.9,$	0.7104	(22.8, 30, 40.8),	$x_{11}, x_{23}, x_{35}, x_{44}, x_{46}$,$x_{62}$
		$23 \le z_{12} \le 41,$		(43.4, 56, 72.2),	
		$37 \le z_{13} \le 51.4$		(10.1, 20, 30.8)	
Aspiration level: (0.7, 0.8, 0.9)					
1	Cost	$15.8 \le z_{11} \le 32.9$	0.8143	(25.9, 34, 47.5),	$x_{11}, x_{13}, x_{44}, x_{46}, x_{55}$,$x_{62}$
		$23 \le z_{12}\le 41,$		(35.3, 47, 62.3),	
		$$32 \le z_{13} \le 56.3$$		(7, 26, 26.8)	
*Shape parameter: (−2, −5, −1)*					
Aspiration level: (0.8, 0.85, 0.7)					
1	Time	$20 \le z_{21} \le 47.3,$	0.8159	(24.9, 33, 46.5),	$x_{13}, x_{14}, x_{46}, x_{51}, x_{55}, x_{62}$
		$29 \le z_{22} \le 59,$		(33.3, 45, 59.4),	
		$$40.7 \le z_{23} \le 75.2$$		(11, 20, 30.8)	
*Shape parameter: (−1, −2, −5)*					
Aspiration level: (0.8, 0.7, 0.75)					
1	Quality	$3.9 \le z_{31}\le 13.1$,	0.7784	(23.7, 30, 41.7),	$x_{12}, x_{15}, x_{23}, x_{34}, x_{46}, x_{61}$
		$12 \le z_{32} \le 22$,		(46.3, 58, 75.1),	
		$22.8\le z_{33} \le 32.8$		(8.1, 18, 28.8)

**Table 6:** Compromised solutions with respect to improvement desired in various objective at confidence level $\alpha =0.5$ with different shape parameter

Case	Obj. function	Bounds	$\lambda$	Objective values	Solution variables
				($Z_{1}, Z_{2}, Z_{3}$)	$x_{ij}$
*Shape parameter: (−5, −1, −2)*					
Aspiration level: (0.8, 0.85, 0.7)					
1	Cost	$19 \le z_{11} \le 36.5$,	0.8255	(30.5, 35, 42.5),	$x_{11}, x_{23}, x_{36}, x_{44}, x_{55}, x_{62}$
		$23 \le z_{12} \le 42$,		(36.5, 43, 51.5),	
		$$28 \le z_{13} \le 50.5$$		(16.5, 22, 28)	
2	Quality	$7.5 \le z_{31} \le 11$,	0.7311	(32.5, 37, 45)	$x_{11}, x_{14}, x_{23}, x_{46}, x_{55}, x_{62}$
		$12 \le z_{32} \le 16$,		(37, 43, 51.5),	
		$$18 \le z_{33} \le 22$$		(9, 14, 20)	
3	Quality	$7.5 \le z_{31} \le 9$,	0.8644	(36, 41, 49.5)	$x_{13}, x_{14}, x_{31}, x_{46}, x_{55}, x_{62}$
		$12 \le z_{32} \le 14$,		(37.5, 43, 51),	
		$$18 \le z_{33} \le 20$$		(7.5, 12, 18)	
Aspiration level: (0.9, 0.7, 0.8)					
1	Cost	$19 \le z_{11} \le 32.5$,	0.7550	(26, 30, 36),	$x_{11}, x_{23}, x_{35}, x_{36}, x_{44}, x_{62}$
		$23 \le z_{12} \le 37$,		(46, 53, 61.5),	
		$$28 \le z_{13} \le 45$$		(18.5, 24, 30)	
2	Quality	$7.5 \le z_{31} \le 9$,	0.8644	(36, 41, 49.5)	$x_{13}, x_{14}, x_{31}, x_{46}, x_{55}, x_{62}$
		$12 \le z_{32} \le 14$,		(37.5, 43, 51),	
		$$18 \le z_{33} \le 20$$		(7.5, 12, 18)	
Aspiration level: (0.7, 0.8, 0.9)					
1	Cost	$19 \le z_{11} \le 36$,	0.8142	(29.5, 34, 41.5),	$x_{11}, x_{23}, x_{24}, x_{46}, x_{55}, x_{62}$
		$$23 \le z_{12} \le 41,$$		(40.5, 47, 56),	
		$$28 \le z_{13} \le 49.5$$		(12.5, 18, 24)	
*Shape parameter: (−2, −5, −1)*					
Aspiration level: (0.8, 0.85, 0.7)					
1	Cost	$19 \le z_{11} \le 25.5$,	0.8187	(21.5, 25, 30.5),	$x_{13}, x_{24}, x_{35}, x_{46}, x_{61}, x_{62}$
		$23 \le z_{12} \le 29$,		(63.5, 71, 81),	
		$$28 \le z_{13} \le 35.5$$		(14.5, 20, 26)	
2	Time	$24 \le z_{21} \le 51.5$,	0.8048	(32.5, 37, 45),	$x_{11}, x_{14}, x_{23}, x_{46}, x_{55}, x_{62}$
		$29 \le z_{22} \le 58$,		(37, 43, 51.5),	
		$$35.5 \le z_{23} \le 67.5$$		(9, 14, 20)	
*Shape parameter: (−1, −2, −5)*					
Aspiration level: (0.7, 0.8, 0.85)					
1	Quality	$7.5 \le z_{31} \le 15$,	0.8124	(28.5, 33, 40.5),	$x_{11}, x_{13}, x_{24}, x_{46}, x_{55}, x_{62}$
		$12 \le z_{32} \le 20$,		(41.5, 48, 56.6),	
		$$18 \le z_{33} \le 26$$		(9, 14, 20)	
2	Quality	$7.5 \le z_{31} \le 11$,	0.7199	(31, 36, 44.5),	$x_{11}, x_{13}, x_{34}, x_{46}, x_{55}, x_{62}$
		$12 \le z_{32} \le 16$,		(41.5, 48, 56.6),	
		$$18 \le z_{33} \le 22$$		(9, 14, 20)	
Aspiration level: (0.8, 0.85, 0.7)					
1	Quality	$7.5 \le z_{31} \le 17$,	0.7848	(26.5, 30, 36.5),	$x_{14}, x_{23}, x_{35}, x_{46}, x_{51}, x_{62}$
		$12 \le z_{32} \le 22$,		(51.5, 58, 67.5),	
		$$18 \le z_{33} \le 28$$		(12.5, 18, 24)

**Table 7:** Compromised solutions with respect to improvement desired in various objective at confidence level $\alpha =0.9$ with different shape parameter

Case	Obj. function	Bounds	$\lambda$	Objective values	Solution variables
				$Z_{1}, Z_{2}, Z_{3}$	$x_{ij}$
*Shape parameter: (−5, −1, −2)*					
Aspiration level: (0.8, 0.85, 0.7)					
1	Cost	$22.2 \le z_{11} \le 40.9$,	0.8578	(33.1, 34, 35.5),	$x_{11}, x_{13}, x_{36}, x_{44}, x_{55}, x_{62}$
		$23 \le z_{12} \le 42$,		(42.7, 44, 45.6),	
		$$24 \le z_{13} \le 43.7$$		(19, 20, 21.2)	
2	Time	$28 \le z_{21} \le 36.9$,	0.7574	(46.7, 48, 49.8),	$x_{14}, x_{31}, x_{36}, x_{52}, x_{55}, x_{62}$
		$29 \le z_{22} \le 38$,		(28, 29, 30.4),	
		$$30.3 \le z_{23} \le 39.5$$		(25, 26, 27.1)	
3	Quality	$11.1 \le z_{31} \le 15$,	0.8681	(40, 41, 42.7),	$x_{13}, x_{14}, x_{31}, x_{46}, x_{55}, x_{62}$
		$12 \le z_{32} \le 16$,		(41.9, 43, 44.6),	
		$13.2 \le z_{33} \le 17.2$		(11.1, 12, 13.2)	
Aspiration level: (0.9, 0.7, 0.8)					
1	Cost	$22.2 \le z_{11} \le 36.1$,	0.7322	(29.3, 30, 31.3),	$x_{14}, x_{23}, x_{35}, x_{36}, x_{51}, x_{62}$
		$23 \le z_{12} \le 37$,		(53.7, 55, 56.8),	
		$24 \le z_{13} \le 38.6$		(20.9, 22, 23.2)	
2	Time	$28 \le z_{21} \le 41.8$,	0.8029	(41.8, 43, 44.7),	$x_{14}, x_{31}, x_{36}, x_{52}, x_{55}, x_{62}$
		$29 \le z_{22} \le 43$,		(31.9, 33, 34.5),	
		$$30.3 \le z_{23} \le 44.7$$		(21, 22, 23.1)	
3	Quality	$11.1 \le z_{31} \le 13$,	0.8681	(40, 41, 42.7),	$x_{13}, x_{14}, x_{31}, x_{46}, x_{55}, x_{62}$
		$12 \le z_{32} \le 14$,		(41.9, 43, 44.6),	
		$13.2 \le z_{33} \le 15.2$		(11.1, 12, 13.2)	
Aspiration level: (0.7, 0.8, 0.9)					
1	Cost	$22.2 \le z_{11} \le 40$,	0.8009	(33.1, 34, 35.4),	$x_{13}, x_{21}, x_{44}, x_{46}, x_{55}, x_{62}$
		$23 \le z_{12} \le 41$,		(47.7, 49, 50.9),	
		$24 \le z_{13} \le 42.7$		(16.9, 18, 19.2)	
*Shape parameter: (−2, −5, −1)*					
Aspiration level: (0.8, 0.85, 0.7)					
1	Cost	$22.2 \le z_{11} \le 33.2$,	0.8215	(25.3, 26, 27.2),	$x_{13}, x_{24}, x_{35}, x_{46}, x_{51}, x_{62}$
		$23 \le z_{12} \le 34$,		(61.6, 63, 64.9),	
		$24 \le z_{13} \le 35.6$		(18.9, 20, 21.2)	
2	Time	$28 \le z_{21} \le 47.8$,	0.7126	(37, 38, 39.5),	$x_{14}, x_{21}, x_{36}, x_{53}, x_{55}, x_{62}$
		$29 \le z_{22} \le 49$,		(39.8, 41, 42.7),	
		$30.3 \le z_{23} \le 50.8$		(22.9, 24, 25.2)	
*Shape parameter: (−1, −2, −5)*					
Aspiration level: (0.7, 0.8, 0.85)					
1	Quality	$11.1 \le z_{31} \le 19,$	0.7972	(33.2, 34, 35.6),	$x_{13}, x_{14}, x_{46}, x_{51}, x_{46}, x_{62}$
		$12 \le z_{32} \le 20$,		(47.8, 49, 50.8),	
		$$13.2 \le z_{33} \le 21.2$$		(13, 14, 15.2)	
Aspiration level: (0.8, 0.7, 0.75)					
2	Quality	$11.1 \le z_{31} \le 21$,	0.8191	(32.1, 33, 34.5),	$x_{11}, x_{13}, x_{24}, x_{46}, x_{55}, x_{62}$
		$12 \le z_{32} \le 22$,		(46.7, 48, 49.7),	
		$$13.2 \le z_{33} \le 23.2$$		(15, 16, 17.2)	

Tables 5, 6 and 7 report the preferred compromise solutions obtained by modifying the upper bounds of various objectives with differing values of confidence level and shape parameters, and differing estimates of aspiration levels for various confidence levels *α*. As shown in the above table, the GA-based hybrid approach helps to improve the TPD by modifying the upper bound of each objective function for particular values of *α* (Gupta and Mehlawat 2014). If the DM is not satisfied with the obtained assignment plans, more assignment plans can be generated by integrating the preference of the DM for various objectives and also altering the various shape parameters.

The GA-based hybrid approach provides flexibility and facilitates the collection of large amounts of information in terms of altering the *α* level and shape parameters in the exponential membership function and providing various scenario analyses to the DM for fuzzy allocation strategy.

## Sensitivity analysis with respect to the number of workers and jobs

Post optimality analysis with respect to the number of workers and jobs is discussed in this section to measure, how the proposed solution method handle FMOAP effectively when new workers and jobs are involve. In this paper, sensitivity analysis is considered by adding the new data of jobs and keeping the workers fixed as given in the article of Gupta and Mehlawat (2014). For $\alpha =0.1$, and the fuzzy input data $l_{i} =3$ and s = 4, solution and assignment plans of FMOAP with extra nine jobs (Job-7 to Job-15) and same six workers are shown in Table 9 with its triangular possibilistic distributions for each objectives.

The computational results are shown in the Table 9 for same six worker and extra nine jobs (6-workers and 9-jobs, 6-workers and 11-jobs, 6-workers and 13-jobs), respectively by taking different estimation of the aspiration levels for each combination of the shape parameters.

We have stated the results by taking different estimation of the aspiration levels for each combination of the shape parameters shown in Table 8.

**Table 8:** Different values of shape parameters and aspiration level

Case	Shape parameter $\left( K_{1}, K_{2}, K_{3} \right)$	Aspiration level $\left( \bar{\mu }_{Z_{1j}} (x),\bar{\mu }_{Z_{2j}} (x), \bar{\mu }_{Z_{3j}} (x)\right)$
Case-1:	(−5, −1, −2)	0.7, 0.8, 0.9
Case-2:	(−5, −1, −2)	0.8, 0.85, 0.7
Case-3:	(−5, −1, −2)	0.9, 0.7, 0.8
Case-4:	(−1, −2, −5)	0.7, 0.8, 0.85
Case-5:	(−1, −2, −5)	0.8, 0.7, 0.75
Case-6:	(−2, −5, −1)	0.8, 0.85, 0.7

**Table 9:** Results summery of sensitivity analysis w.r.t number of jobs at $\alpha = 0.1$

No. of jobs	Case	*W*	Membership values $\left( \mu _{Z_{1j}}, \mu _{Z_{2j}}, \mu _{Z_{3j}} \right)$	Objective values $\left( Z_{1}, Z_{2}, Z_{3} \right)$	Optimum allocations					
					$x_{1j}$	$x_{2j}$	$x_{3j}$	$x_{4j}$	$x_{5j}$	$x_{6j}$
Jobs-9	1	0.8235	(0.9574, 0.9567, 0.9287) (0.8549, 0.8367, 0.8235) (0.9184, 0.9124, 0.9152)	(49.8, 66, 90.3) (47.9, 65, 85.7) (14.4, 27, 42.3)	${x_{11}}$ ${x_{13}}$ ${x_{14}}$		$x_{37}$	${x_{48}}$ ${x_{49}}$	${x_{52}}$ ${x_{55}}$ ${x_{56}}$	
	2	0.8716	(0.9148, 0.9170, 0.8824) (0.8899, 0.8757, 0.8716) (0.9270, 0.9124, 0.9066)	(55.9, 73, 96.4) (43.8, 60, 78.9) (13.5, 27, 43.2)	${x_{11}}$ ${x_{14}}$ ${x_{19}}$		$x_{38}$		${x_{55}}$ ${x_{56}}$	${x_{62}}$ ${x_{63}}$ ${x_{67}}$
	3	0.8436	(0.9626, 0.9644, 0.9431) (0.8713, 0.8447, 0.8436) (0.8882, 0.8936, 0.8957)	(48.7, 64, 87.4) (46, 64, 82.9) (17.3, 29, 44.3)	${x_{11}}$ ${x_{13}}$ ${x_{14}}$		$x_{36}$	${x_{48}}$ ${x_{49}}$	${x_{52}}$ ${x_{55}}$	$x_{67}$
	4	0.8036	(0.8671, 0.8829, 0.8684) (0.8233, 0.8243, 0.8036) (0.9515, 0.9442, 0.9423)	(40.4, 53, 71) (60.8, 77, 100.4) (24.6, 39, 54.3)	${x_{11}}$ ${x_{14}}$ ${x_{17}}$	$x_{23}$	${x_{32}}$ ${x_{35}}$ ${x_{38}}$	${x_{46}}$ ${x_{49}}$		
	5	0.7452	(0.7856, 0.7832, 0.7452) (0.9174, 0.8998, 0.8813) (0.9740, 0.9709, 0.9709)	(45.7, 61, 81.7) (47, 65, 87.5) (19.5, 33, 48.3)	${x_{11}}$ ${x_{13}}$		$x_{38}$	${x_{44}}$ ${x_{46}}$ ${x_{49}}$	${x_{52}}$ ${x_{55}}$	$x_{67}$
	6	0.8434	(0.8763, 0.8807, 0.8434) (0.9665, 0.9623, 0.9527) (0.8902, 0.8552, 0.8466)	(44.6, 59, 80.6) (59, 77, 101.3) (12.6, 27, 43.2)	$x_{13}$	${x_{21}}$ ${x_{24}}$	$x_{37}$	${x_{46}}$ ${x_{48}}$ ${x_{49}}$	$x_{55}$	$x_{62}$
Jobs-11	1	0.8528	(0.9490, 0.9456, 0.9283) (0.8735, 0.8685, 0.8528) (0.9241, 0.9203, 0.9201)	(63.3, 84, 111) (57.2, 77, 102.2) (17.7, 33, 51.9)	${x_{11}}$ ${x_{13}}$ ${x_{14}}$		$x_{38}$	${x_{46}}$ ${x_{411}}$	${x_{52}}$ ${x_{55}}$ ${x_{59}}$	${x_{67}}$ ${x_{610}}$
	2	0.8516	(0.9503, 0.9567, 0.9406) (0.8655, 0.8552, 0.8516) (0.9241, 0.9203, 0.9201)	(63, 81, 108) (58.3, 79, 102.4) (17.7, 33, 51.9)	${x_{13}}$ ${x_{14}}$ ${x_{111}}$	$x_{210}$	$x_{31}$	${x_{46}}$ ${x_{48}}$ ${x_{49}}$	${x_{52}}$ ${x_{55}}$	$x_{67}$
	3	0.8706	(0.9296, 0.9456, 0.9359) (0.8885, 0.8881, 0.8706) (0.8995, 0.8908, 0.8883)	(66.9, 84, 109.2) (55.1, 74, 99.2) (20.8, 37, 55.9)	${x_{13}}$ ${x_{14}}$ ${x_{111}}$	$x_{210}$	$x_{31}$	${x_{46}}$ ${x_{47}}$	${x_{55}}$ ${x_{58}}$	${x_{62}}$ ${x_{69}}$
	4	0.8304	(0.8498, 0.8519, 0.8304) (0.8409, 0.8402, 0.8461) (0.9651, 0.9609, 0.9587)	(50.9, 68, 90.5) (72.3, 93, 116.4) (27.9, 45, 63.9)	${x_{13}}$ ${x_{111}}$	$x_{21}$	${x_{36}}$ ${x_{37}}$ ${x_{38}}$	${x_{44}}$ ${x_{45}}$ ${x_{49}}$		${x_{62}}$ ${x_{610}}$
	5	0.8105	(0.9074, 0.9081, 0.8874) (0.8210, 0.8221, 0.8105) (0.9438, 0.9354, 0.9336)	(45.8, 62, 83.6) (75.3, 96, 123) (33, 51, 69)	${x_{13}}$ ${x_{111}}$	$x_{24}$	$x_{35}$	${x_{46}}$ ${x_{49}}$	${x_{51}}$ ${x_{52}}$ ${x_{58}}$	${x_{67}}$ ${x_{610}}$
	6	0.8093	(0.9096, 0.9167, 0.9066) (0.9590, 0.9615, 0.9575) (0.8577, 0.8236, 0.8093)	(50.8, 67, 88.6) (76.2, 96, 123) (19, 37, 56.8)	${x_{14}}$ ${x_{111}}$	${x_{21}}$ ${x_{23}}$	${x_{35}}$ ${x_{37}}$	${x_{46}}$ ${x_{48}}$ ${x_{49}}$		${x_{62}}$ ${x_{610}}$
Jobs-13	1	0.8118	(0.9098, 0.9265, 0.9127) (0.8518, 0.8248, 0.8118) (0.9182, 0.9110, 0.9014)	(83.4, 105, 137.4) (71, 98, 128.6) (24.1, 43, 66.4)	${x_{13}}$ ${x_{113}}$	${x_{210}}$ ${x_{211}}$	${x_{31}}$ ${x_{36}}$	${x_{44}}$ ${x_{48}}$ ${x_{49}}$	${x_{55}}$ ${x_{512}}$	${x_{62}}$ ${x_{67}}$
	2	0.8636	(0.8734, 0.8705, 0.8837) (0.9086, 0.9056, 0.9031) (0.8824, 0.9713, 0.8636)	(87.9, 114, 142.8) (61.4, 83, 110) (29.2, 49, 71.5)	${x_{13}}$ ${x_{14}}$ ${x_{113}}$	${x_{21}}$ ${x_{26}}$ ${x_{29}}$	${x_{38}}$ ${x_{312}}$	${x_{45}}$ ${x_{47}}$ ${x_{411}}$	$x_{52}$	$x_{610}$
	3	0.8170	(0.9598, 0.9672, 0.9550) (0.8469, 0.8305, 0.8170) (0.9109, 0.8985, 0.8872)	(73.2, 93, 125.4) (71.8, 97, 127.6) (25.2, 45, 68.4)	${x_{11}}$ ${x_{14}}$ ${x_{111}}$	${x_{23}}$ ${x_{210}}$	${x_{36}}$ ${x_{37}}$	${x_{48}}$ ${x_{412}}$	${x_{55}}$ ${x_{513}}$	${x_{62}}$ ${x_{69}}$
	4	0.8260	(0.8260, 0.8322, 0.8364) (0.8727, 0.8786, 0.8586) (0.9708, 0.9609, 0.9542)	(52.9, 70, 89.8) (67.1, 86, 113.9) (26.1, 45, 64.8)	${x_{13}}$ ${x_{111}}$	$x_{21}$	${x_{36}}$ ${x_{37}}$ ${x_{38}}$	${x_{44}}$ ${x_{45}}$ ${x_{49}}$		${x_{62}}$ ${x_{610}}$
	5	0.8296	(0.8380, 0.8421, 0.8296) (0.8778, 0.8786, 0.8630) (0.9830, 0.9773, 0.9740)	(51.9, 69, 90.6) (66.2, 86, 113) (21, 39, 58.8)	${x_{13}}$ ${x_{111}}$	$x_{21}$	$x_{38}$	${x_{46}}$ ${x_{47}}$ ${x_{49}}$	$x_{55}$	${x_{62}}$ ${x_{67}}$ ${x_{610}}$
	6	0.8186	(0.8438, 0.8540, 0.8352) (0.9749, 0.9726, 0.9684) (08619, 0.8347, 0.8186)	(74.3, 95, 125.6) (82.8, 108, 139.5) (30.3, 51, 73.5)	${x_{15}}$ ${x_{111}}$ ${x_{113}}$	${x_{23}}$ ${x_{24}}$	$x_{37}$	${x_{46}}$ ${x_{48}}$	${x_{51}}$ ${x_{512}}$	${x_{62}}$ ${x_{69}}$ ${x_{610}}$

The computational results and its corresponding assignment plans of sensitivity analysis (15-jobs and 6-workers) are presented in the Table 9 at $\alpha =0.1$ for different shape parameter and aspiration level.

Moreover, this paper presents a sensitivity analysis by adding the new data of workers (Worker-7 to Worker-9) and keeping the jobs (Job-1 to Job-15) as given in the article of Gupta and Mehlawat (2014). For $\alpha =0.1$, and the fuzzy input data $l_{i} =3$ and s = 4, solution and assignment plans of FMOAP with extra workers (worker-7 to worker-9) and fifteen jobs are shown in Table 11 with its triangular possibilistic distributions for each objectives.

The computational results are shown in the Table 11 or additional worker and 15 jobs (7-workers and 15-jobs, 8-workers and 15-jobs, 9-workers and 15-jobs) respectively by taking different estimation of the aspiration levels for each combination of the shape parameters.

We have stated the results by taking different estimation of the aspiration levels for each combination of the shape parameters shown in Table 10.

**Table 10:** Different values of shape parameters and aspiration level

Case	Shape parameter $\left( K_{1}, K_{2}, K_{3} \right)$	Aspiration level $\left( \bar{\mu }_{Z_{1j}} (x),\bar{\mu }_{Z_{2j}} (x), \bar{\mu }_{Z_{3j}} (x)\right)$
Case-1	(−5, −1, −2)	0.8, 0.85, 0.7
Case-2	(−5, −1, −2)	0.9, 0.7, 0.8
Case-3	(−1, −2, −5)	0.7, 0.8, 0.85
Case-4	(−1, −2, −5)	0.8, 0.7, 0.75
Case-5	(−2, −5, −1)	0.8, 0.85, 0.7

**Table 11:** Results summery of sensitivity analysis w.r.t number of workers at $\alpha = 0.1$

No. of worker	Case	$\lambda$	$\mu _{ij}$	Objective values	Optimum allocations $x_{ij}$
Workers-7	1	0.7480	(0.8986, 0.9066, 0.8964) (0.9182, 0.9036, 0.8840) (0.8606, 0.8387, 0.7480)	(105, 132, 168.9) (68, 95, 131.9) (36.7, 61, 87.1)	${x_{13}, x_{112}, x_{115}, x_{24}, x_{210},}$ ${x_{31}, x_{36}, x_{38}, x_{411}, x_{413},}$ ${x_{55}, x_{59}, x_{62}, x_{67}, x_{614}}$
	2	0.8067	(0.9198, 0.9280, 0.9414) (0.8768, 0.8616, 0.8491) (0.9052, 0.8875, 0.8067)	(100.9, 127, 156.7) (76.1, 104, 140) (29.6, 53, 79.1)	${x_{11}, x_{14}, x_{111}, x_{23}, x_{210},}$ ${x_{215}, x_{38}, x_{312}, x_{45}, x_{52},}$ ${ x_{56}, x_{513}, x_{69}, x_{614}, x_{77}}$
	3	0.7303	(0.7716, 0.7433, 0.7303) (0.8944, 0.8933, 0.8805) (0.9648, 0.9577, 0.9162)	(85.2, 114, 148.2) (84.9, 111, 148.8) (40.7, 65, 91.1)	${x_{12}, x_{18}, x_{112}, x_{23}, x_{24},}$ ${x_{36}, x_{37}, x_{314}, x_{49}, x_{411},}$ ${x_{413}, x_{52}, x_{610}, x_{615}, x_{75}}$
	4	0.8131	(0.8131, 0.8202, 0.8304) (0.8362, 0.8263, 0.8287) (0.9597, 0.9523, 0.9070)	(80.6, 104, 133.7) (98.3, 128, 163.1) (42.7, 67, 93.1)	${x_{13}, x_{19}, x_{111}, x_{21}, x_{213},}$ ${x_{32}, x_{35}, x_{314}, x_{44}, x_{46},}$ ${x_{47}, x_{58}, x_{512}, x_{515}, x_{610}}$
	5	0.7539	(0.8179, 0.8122, 0.8251) (0.9353, 0.9320, 0.9332) (0.8937, 0.8652, 0.7539)	(90, 117, 147.6) (113.4, 144, 180.9) (23.6, 47, 74)	${x_{19}, x_{111}, x_{215}, x_{33}, x_{34},}$ ${x_{314}, x_{45}, x_{48}, x_{51}, x_{512},}$ ${x_{513}, x_{62}, x_{610}, x_{76}, x_{77}}$
Workers-8	1	0.7932	(0.9092, 0.9083, 0.9110) (0.8911, 0.8836, 0.8578) (0.8482, 0.8187, 0.7932)	(108.2, 137, 170.3) (71.8, 97, 134.8) (37.8, 63, 90)	${x_{111}, x_{114}, x_{24}, x_{215}, x_{38},}$ ${x_{46}, x_{47}, x_{51}, x_{63}, x_{69},}$ ${x_{710}, x_{712}, x_{713}, x_{82}, x_{85}}$
	2	0.7752	(0.9492, 0.9511, 0.9385) (0.8113, 0.7849, 0.7752) (0.9389, 0.9266, 0.9225)	(97.9, 124, 161.8) (87.4, 118, 154) (21.4, 43, 69.1)	${x_{11}, x_{13}, x_{112}, x_{210}, x_{34},}$ ${x_{314}, x_{46}, x_{47}, x_{411}, x_{58},}$ ${x_{513}, x_{62}, x_{615}, x_{85}, x_{89}}$
	3	0.7408	(0.7504, 0.7408, 0.7626) (0.8687, 0.8702, 0.8712) (0.9754, 0.9685, 0.9638)	(91, 118, 146.8) (90.8, 116, 149.3) (34.7, 59, 85.1)	${x_{111}, x_{112}, x_{21}, x_{23}, x_{215},}$ ${x_{314}, x_{45}, x_{48}, x_{49}, x_{52},}$ ${x_{59}, x_{610}, x_{84}, x_{87}, x_{813}}$
	4	0.8069	(0.8112, 0.8181, 0.8096) (0.8719, 0.8666, 0.8621) (0.9698, 0.9604, 0.9537)	(83.6, 107, 139.4) (90, 117, 152.1) (37.8, 63, 89.1)	${x_{14}, x_{111}, x_{115}, x_{29}, x_{38},}$ ${x_{314}, x_{46}, x_{412}, x_{55}, x_{513},}$ ${x_{62}, x_{610}, x_{77}, x_{83}}$
	5	0.8168	(0.8434, 0.8352, 0.8423) (0.9572, 0.9560, 0.9540) (0.8645, 0.8347, 0.8168)	(90, 117, 147.6) (104.2, 132, 168.9) (25.6, 49, 76)	${x_{14}, x_{111}, x_{215}, x_{314}, x_{46},}$ ${x_{48}, x_{53}, x_{55}, x_{59}, x_{61},}$ ${x_{610}, x_{612}, x_{77}, x_{82}, x_{813}}$
Workers-9	1	0.7975	(0.9192, 0.8991, 0.8994) (0.9197, 0.9064, 0.8898) (0.8401, 0.8158, 0.7975)	(108.8, 143, 177.2) (65.7, 90, 125.1) (37.6, 61, 87.1)	${x_{16}, x_{111}, x_{113}, x_{28}, x_{29},}$ ${x_{215}, x_{31}, x_{37}, x_{44}, x_{52},}$ ${x_{73}, x_{85}, x_{812}, x_{910}, x_{914}}$
	2	0.8208	(0.9371, 0.9380, 0.9279) (0.8636, 0.8265, 0.8208) (0.9149, 0.8936, 0.8810)	(104.1, 132, 168.9) (77.4, 108, 142.2) (23.6, 47, 74)	${x_{113}, x_{21}, x_{210}, x_{312}, x_{44},}$ ${x_{48}, x_{411}, x_{55}, x_{62}, x_{67},}$ ${x_{714}, x_{89}, x_{93}, x_{96}, x_{915}}$
	3	0.7298	(0.7346, 0.7429, 0.7298) (0.9093, 0.8960, 0.8809) (0.9524, 0.9381, 0.9258)	(94.8, 120, 154.2) (80, 107, 144.8) (43.8, 69, 95.1)	${x_{14}, x_{112}, x_{115}, x_{23}, x_{29},}$ ${x_{211}, x_{38}, x_{314}, x_{46}, x_{51},}$ ${x_{62}, x_{75}, x_{713}, x_{97}, x_{910}}$
	4	0.7653	(0.8351, 0.8271, 0.8083) (0.8056, 0.7922, 0.7653) (0.9626, 0.9497, 0.9435)	(81.8, 107, 141.2) (105.2, 134, 176.3) (39.8, 65, 90.2)	${x_{17}, x_{18}, x_{111}, x_{23}, x_{26},}$ ${x_{34}, x_{314}, x_{45}, x_{49}, x_{413},}$ ${x_{52}, x_{512}, x_{61}, x_{910}, x_{915}}$
	5	0.7615	(0.8495, 0.8517, 0.8607) (0.9718, 0.9657, 0.9585) (0.8275, 0.7847, 0.7615)	(90.8, 111.6, 145.7) (94.3, 124, 164.5) (28.7, 53, 80)	${x_{111}, x_{38}, x_{314}, x_{44}, x_{46},}$ ${x_{413}, x_{55}, x_{61}, x_{610}, x_{77},}$ ${x_{89}, x_{812}, x_{815}, x_{92}, x_{93}}$

The computational results and the corresponding assignment plans obtained through sensitivity analysis (15 jobs and 9 employees) are presented in Table 11 at $\alpha =0.1$ for various shape parameters and estimated aspiration levels. Moreover, as previously discussed, if the DM is not satisfied with the obtained compromise solution, more solutions can be obtained by improving an individual objective function as per the DM’s preference.

Thus, the sensitivity analysis reveals that the developed solution approach can handle the FMOAP successfully and proficiently when an additional employee and \or job is considered. In addition, if the DM is not satisfied with the obtained assignment plans, more assignment plans can be generated by changing the values of the shape parameters in the exponential membership function (Gupta and Mehlawat 2013, 2014; Tailor and Dhodiya 2016a).

## Comparison

Table 12 shows comparison between obtained solutions by GA based hybrid approach using exponential membership function with different approaches at $\alpha = 0.1$.

**Table 12:** comparison between obtained solutions by GA based hybrid approach using exponential membership function with different approaches at $\alpha = 0.1$

Shape parameter	Aspiration level	Hannan (1981)	Lai and Hwang (1992)	Yager (1981)	Gupta and Mehlawat (2014)	Proposed hybrid approach
		Max $\lambda$	Max $\lambda$	Max $\lambda$	Max $\lambda$	Max $\lambda$
(−5, −1, −2)	0.8, 0.85, 0.7	0.67342	0.53334	0.642109	0.695652	0.8954
(−5, −1, −2)	0.9, 0.7, 0.8					0.8527
(−5, −1, −2)	0.7, 0.8, 0.9					0.8611
(−2, −5, −1)	0.8, 0.85, 0.7					0.9115
(−2, −5, −1)	0.9, 0.75, 0.8					0.8667
(−1, −2, −5)	0.7, 0.8, 0.85					0.7799
(−1, −2, −5)	0.8, 0.7, 0.75					0.8240

## Conclusion

The GA-based hybrid approach provided the solution for the FMOAP by using the fuzzy exponential membership function with some realistic constraints to optimize the optimistic, most likely, and pessimistic scenarios of fuzzy objective functions with TPD. Moreover, the developed hybrid approach provided flexibility for the DM in terms of the various choices in aspiration levels, shape parameters, upper bound improvement and also provided more effective assignment plans.
